# Differential regulation by CD47 and thrombospondin-1 of extramedullary erythropoiesis in mouse spleen

**DOI:** 10.1101/2023.09.28.559992

**Published:** 2023-09-28

**Authors:** Rajdeep Banerjee, Thomas J. Meyer, Margaret C. Cam, Sukhbir Kaur, David D. Roberts

**Affiliations:** 1 Laboratory of Pathology, Center for Cancer Research, National Cancer Institute, National Institutes of Health, Bethesda, MD, USA; 2 CCR Collaborative Bioinformatics Resource, Office of Science and Technology Resources, National Cancer Institute, National Institutes of Health, Bethesda, MD, USA

## Abstract

Extramedullary erythropoiesis is not expected in healthy adult mice, but erythropoietic gene expression was elevated in lineage-depleted spleen cells from *cd47*^−/−^ mice. Expression of several genes associated with early stages of erythropoiesis was elevated in mice lacking CD47 or its signaling ligand thrombospondin-1, consistent with previous evidence that this signaling pathway inhibits expression of multipotent stem cell transcription factors in spleen. In contrast, cells expressing markers of committed erythroid progenitors were more abundant in *cd47*^−/−^ spleens but significantly depleted in *thbs1*^−/−^ spleens. Single cell transcriptome and flow cytometry analyses indicated that loss of CD47 is associated with accumulation and increased proliferation in spleen of Ter119^−^CD34^+^ progenitors and Ter119^+^CD34^−^ committed erythroid progenitors with elevated mRNA expression of Kit, Ermap, and Tfrc, Induction of committed erythroid precursors is consistent with the known function of CD47 to limit the phagocytic removal of aged erythrocytes. Conversely, loss of thrombospondin-1 delays the turnover of aged red blood cells, which may account for the suppression of committed erythroid precursors in *thbs1*^−/−^ spleens relative to basal levels in wild type mice. In addition to defining a role for CD47 to limit extramedullary erythropoiesis, these studies reveal a thrombospondin-1-dependent basal level of extramedullary erythropoiesis in adult mouse spleen.

## Introduction

CD47 is a counter-receptor for signal-regulatory protein-α (SIRPα)^1^ and a component of a supramolecular membrane signaling complex for thrombospondin-1 that contains specific integrins, heterotrimeric G proteins, tyrosine kinase receptors, exportin-1, and ubiquilins.^2–5^ CD47 binding to SIRPα on macrophages induces inhibitory signaling mediated by its cytoplasmic immunoreceptor tyrosine-based inhibition motifs that recruit and activate the tyrosine phosphatases SHP-1 and SHP-2.^1^ Loss of this inhibitory signaling results in rapid splenic clearance of *cd47*^−/−^ mouse red blood cells (RBC) when transfused into a wild type (WT) recipient.^6^ The species-specificity of CD47/SIRPα binding constitutes a barrier to interspecies blood transfusion and hematopoietic reconstitution.^7,8^

CD47 forms nanoclusters on young RBC with limited binding to thrombospondin-1.^9^ CD47 abundance decreases on aged RBCs, but CD47 on aging RBC forms larger and more dense clusters with increased ability to bind thrombospondin-1. CD47 on aging RBC also adopts an altered conformation.^10^ Exposure of aged RBC to thrombospondin-1 further increased the size of CD47 clusters via a lipid raft-dependent mechanism. Conversely, CD47 cluster formation was limited on *thbs1*^−/−^ mouse RBC and associated with significantly increased RBC lifespan.^9^

Liver and spleen are the main hematopoietic organs during embryonic development, whereas bone marrow assumes that responsibility after birth.^11^ Induction of extramedullary hematopoiesis in adult spleen can compensate for pathological conditions that compromise hematopoiesis in bone marrow.^12^ CD47 is highly expressed on proliferating erythroblasts during stress-induced erythropoiesis, and antibodies blocking either CD47 or SIRPα inhibited the required transfer of mitochondria from macrophages to developing erythroblasts in erythroblastic islands.^13^ Notably, treatment with a CD47 antibody enhanced splenomegaly in the anemic stress model. Consistent with the absence of inhibitory SIRPα signaling that limits clearance of aged RBC,^14,15^ *cd47*^−/−^ mice derived using CRISPR/Cas9 exhibited hemolytic anemia and splenomegaly.^16^ Conversely, CD47-dependent thrombospondin-1 signaling regulates the differentiation of multipotent stem cells in a stage-specific manner,^17–19^ and both *thbs1*^−/−^ and *cd47*^−/−^ mouse spleens have more abundant Sox2^+^ stem cells and higher mRNA expression of the multipotent stem cell transcription factors Myc, Sox2, Oct4, and Klf4.^17^ Therefore, both thrombospondin-1- and SIRPα-dependent CD47 signaling could alter erythropoiesis and contribute to spleen enlargement. Here, we utilized flow cytometry combined with bulk and single cell transcriptomics to examine extramedullary hematopoiesis in *cd47*^−/−^ and *thbs1*^−/−^ mice, which revealed cooperative and opposing roles for CD47 and thrombospondin-1 to limit extramedullary erythropoiesis in spleen.

## RESULTS

### Upregulation of erythroid precursors in *cd47*^−/−^ mouse spleen

**Figure 1:**
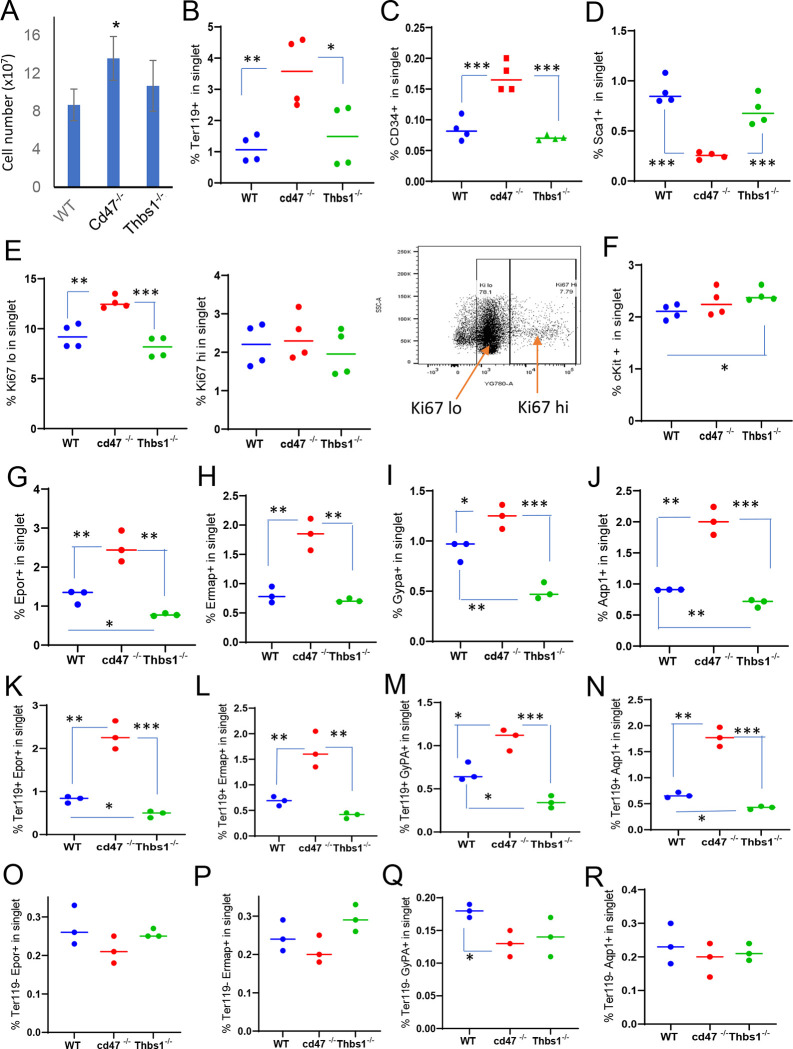
Effects of *Cd47* or *Thbs1* gene disruption on spleen cell numbers and content of cells expressing erythropoietic precursor markers or the proliferation marker Ki67. (A) Total spleen cell numbers in WT, *cd47*^−/−^ and *thbs1*^−/−^ C57BL/6 mice determined after lysis of RBC (mean±SEM, n =3). Flow cytometry was performed to analyze gated singlet spleen cells stained with Ter119 antibody (B), CD34 antibody (C), Sca1 antibody (D), Ki67 antibody with the indicated gating for high and low expression (E), cKit antibody (F), Epor antibody (G), Ermap antibody (H), Gypa antibody (I), or Aqp1 antibody (J). Further analysis of Epor (K,O), Ermap (L,P), Gypa (M,Q), and Aqp1 expression (N,R) was performed after gating for Ter119 expression. The percentages of cells positive for the indicated surface markers are presented (n = 3 or 4) P-values were determined using a two-tailed t test for two-samples assuming equal variances in GraphPad Prism. * = p<0.05, ** = p<0.01, *** = p<0.001

We confirmed the previously reported spleen enlargement in *cd47*^−/−^ mice,^18,20^ but did not observe significant spleen enlargement in *thbs1*^−/−^ mice (supplemental Figure 1A). Enlargement of *cd47*^−/−^ spleens could result from increased phagocytic clearance of RBC, as reported in *cd47*^−/−^ and *sirpa*^−/−^ mice treated with CpG^21^ and aging *cd47*^−/−^ mice,^16^ or increased cell numbers could result from the increased stem cell abundance in *cd47*^−/−^ spleens.^17^ The spleen enlargement was associated with a significantly higher total spleen cell number in a single cell suspension after RBC lysis in *cd47*^−/−^ mice compared to WT and *thbs1*^−/−^ mice (Figure 1A).

Our previous analysis of lineage-negative cells from WT and *cd47*^−/−^ spleens identified an increased abundance of NK cell precursors in *cd47*^−/−^ spleens.^18^ Analysis of bulk RNAseq data of naïve WT and *cd47*^−/−^ spleen cells depleted for proerythroblasts through mature erythrocytes using the antibody Ter-119^22^ and for cells bearing CD4, CD11b, CD11c, CD19, CD45R, CD49b, CD105, MHC Class II, and TCRγ/δ (Lin^−^CD8^+^) unexpectedly showed strong enrichment of a heme metabolism gene signature (supplemental Figure 1C), markers of stress-induced erythropoiesis^23,24^ and adult definitive erythropoiesis^25^ in the lineage-depleted *cd47*^−/−^ relative to the corresponding cells from WT spleens (Table 1, supplemental Figure 1B,D). Trim10 mRNA, which encodes an erythroid-specific RING finger protein required for terminal erythroid differentiation,^26^ was elevated 50-fold. Higher Mki67 mRNA expression suggested increased proliferation among Lin^−^ *cd47*^−/−^ spleen cells, which also expressed elevated mRNA levels of the major erythroid transcription factor Gata1.^27^ Apart from increased Kit mRNA expression, however, mRNA expression of markers for multipotent erythroid progenitors including Anpep (CD13), Cd33, Sca1 and Gata2 was not elevated in the absence of CD47. These data suggested preferential accumulation of committed erythroid progenitors rather than multipotent erythroid progenitors in the *cd47*^−/−^ spleens. CD47 regulates activities of the nuclear transport protein exportin-1,^5^ and Xpo1 mRNA was also increased in *cd47*^−/−^ cells (Table 1). Exportin-1 is a Gata1 transcriptional target and promotes terminal erythroid differentiation by maintaining Gata1 in the nucleus,^28^ which suggested a potential mechanism by which loss of CD47 could increase erythropoiesis.

To further characterize CD47-dependent spleen cells and the relevance of thrombospondin-1, single cell suspensions of depleted of mature RBC were analyzed using flow cytometry for expression of erythropoiesis-related cell surface and proliferation markers (Figure 1B–J, supplemental Figure 2). The percentages of Ter119^+^ cells in singlet cells from *cd47*^−/−^ spleens was significantly higher than in WT or *thbs1*^−/−^ spleens (p = 0.0061 and 0.0322 respectively, Figure 1B). CD34 is expressed on multipotent through the CFU-E erythroid progenitors and was expressed a significantly higher percentage on *cd47*^−/−^ versus WT or *thbs1*^−/−^ cells (p = 0.0008 and 0.0001 respectively, Figure 1C), whereas the multipotent progenitor marker Sca1 was expressed in a smaller percentage of *cd47*^−/−^ versus WT or *thbs1*^−/−^ spleen cells (p = 0.0001 and 0.0009 respectively, Figure 1D).

A higher percentage of *cd47*^−/−^ cells expressed the proliferation marker Ki67 at low but not high levels compared to WT or *thbs1*^−/−^ spleen cells (p = 0.0024 and 0.0003 respectively, Figure 1E). c-Kit signaling is crucial for normal hematopoiesis and is expressed in multipotent progenitors through CFU-E,^29,30^ but the percentage of cKit positive cells was higher only in *thbs1*^−/−^ spleen cells (Figure 1F).

Erythrocyte lineage markers including Ermap, glycophorin A (Gypa), Epor and Aqp1 are established markers of stress-induced extramedullary erythropoiesis.^24^ The erythropoietin receptor (Epor), which is expressed in CFU-E through proerythroblasts, was expressed in significantly more *cd47*^−/−^ versus WT spleen cells (p = 0.0077) but in significantly fewer *thbs1*^−/−^ spleen cells (p = 0.0017 Figure 1G). Cells expressing the major RBC membrane glycoprotein Gypa, which accumulates in erythroblasts, Ermap, and Aqp1 showed similar significant increases in *cd47*^−/−^ spleen cells, whereas only Gyp1^+^ and Aqp1^+^ cells were significantly decreased in *thbs1*^−/−^ spleen cells (Figure 1H, I, J). Consistent with their known induction kinetics, most of the cells expressing these markers were Ter119^+^, and the alterations in their abundance observed in *cd47*^−/−^ and *thbs1*^−/−^ spleens were restricted to Ter119^+^ cells (Figure 1K–R). These results indicate an increased abundance of committed erythroid progenitors spanning CD34^+^ progenitors through reticulocytes in the *cd47*^−/−^ spleen but depletion of the earlier Sca1^+^ multipotent progenitors. Consistent with the decreased turnover of *thbs1*^−/−^ RBC,^9^ cells expressing the committed erythroid markers Gypa, Epor, and Aqp1 were depleted in *thbs1*^−/−^ spleens.

Erythropoietin binding to Epor on early erythroid precursor cells stimulates their survival, proliferation, and differentiation by inducing the master transcriptional regulators Tal1, Gata1, and Klf1.^31^ Tal1, Gata1, and Klf1, and Epor mRNAs were strongly up-regulated in *cd47*^−/−^ relative to WT spleens (Table 1). The increased proliferative response in the *cd47*^−/−^ cells is consistent with a significant increase in Epor^+^ Ter119^+^ *cd47*^−/−^ cells, contrasting with a significant decreased in their abundance in Ter119^+^ *thbs1*^−/−^ cells (Figure 1K). Epor expression was minimal and unchanged in the Ter119^−^ population (Figure 1O), indicating that the Ter119 antigen is expressed in erythroid precursors that are responsive to erythropoietin. These data are consistent with increased extramedullary erythropoiesis in *cd47*^−/−^ and suppressed extramedullary erythropoiesis relative to WT in *thbs1*^−/−^ spleens.

### CD47-dependence of erythropoietic markers in Ter119^+^ and Ter119^−^ cells

**Figure 2:**
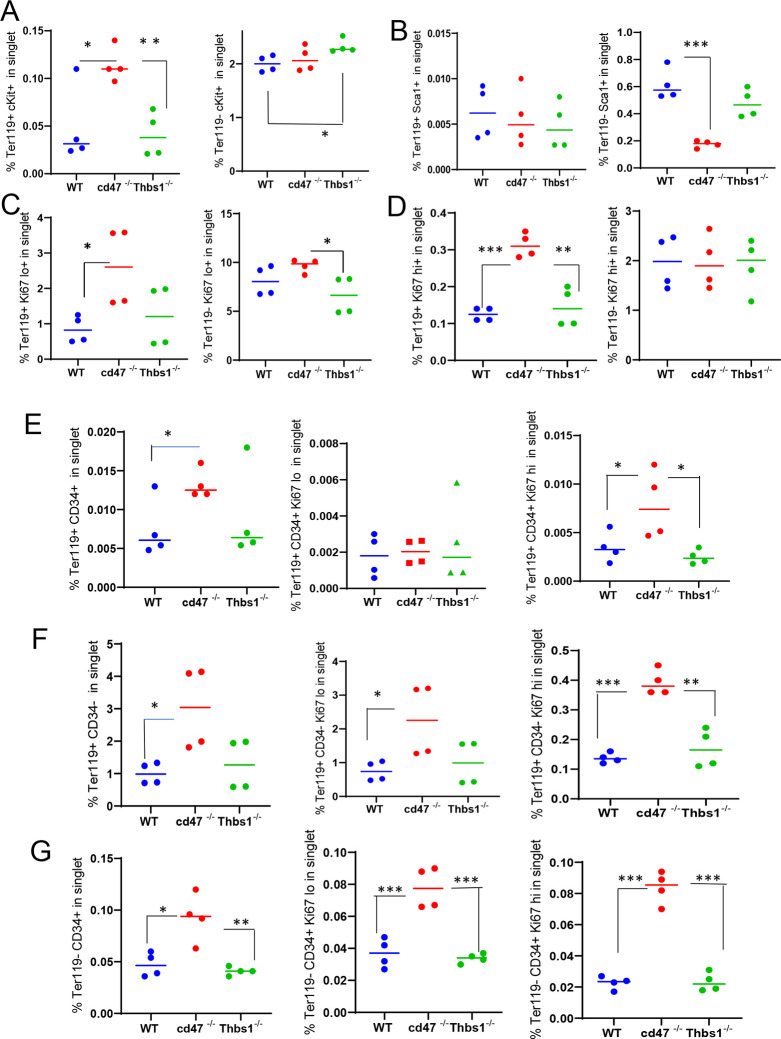
Effects of *Cd47* or *Thbs1* gene disruption on the percentage of Ter119^+^ and Ter119^−^ spleen cells expressing markers of multipotent and committed erythroid precursors and cell proliferation. Spleen cells isolated from WT, *cd47*^−/−^ and *thbs1*^−/−^ mice were costained with Ter119 antibody along with cKit, Ki67, Sca1 Ermap, Gypa, Epor or Aqp1 antibodies and acquired on an LSRFortessa SORP. After gating for singlet cells, the percentages of Ter119^+^ and Ter119^−^ cells positive for stem cell markers cKit (A) and Sca1 (B), high or low levels of the proliferation marker Ki67 (C,D), were compared among WT, *cd47*^−/−^ and *thbs1*^−/−^ mouse spleens (n = 3–4). The proliferation of CD34^+^ and CD34^−^ populations of Ter119^+^ spleen cells from WT, *cd47*^−/−^, *thbs1*^−/−^ mice was evaluated by staining with CD34, Ter119 and Ki67 antibodies. Ter119^+^CD34^+^ cells (E), Ter119^+^CD34^−^ cells (F), and Ter119^−^CD34^+^ cells (G) were also quantified (left panels) and analyzed for the proliferation marker Ki67 (center and right panels). P-values were determined using a two-tailed t test for two-samples assuming equal variances in GraphPad Prism. * = p<0.05, ** = p<0.01, *** = p<0.001

The Ter119 antibody recognizes an antigen highly expressed in mouse proerythroblasts through mature erythrocytes.^22^ Although Ter119 is a widely used erythroid lineage marker, its epitope does not map to a specific protein^22^ and requires 9-O-acetylation of sialic acids that are present on several RBC glycoproteins.^32^ Although no effect of CD47 on the abundance of cKit^+^ cells was detected (Figure 1F), Kit mRNA was significantly enriched in lineage-negative *cd47*^−/−^ spleen cells (Table 1). To resolve this discrepancy, the percentage of cKit^+^ cells was assessed in Ter119^+^ and Ter119^−^ populations (Figure 2A). A higher percentage of the *cd47*^−/−^ cells were Ter119^+^cKit^+^. Consistent with Fig 1F and the elevation of multipotent stem cells in *thbs1*^−/−^ spleen,^17^ Ter119^−^cKit+ cells were elevated in *thbs1*^−/−^ spleens. Consistent with the bulk RNA sequencing data, Sca1^+^ cells in spleen did not differ in the Ter119^+^ cells and were less abundant in Ter119^−^ cells from *cd47*^−/−^ spleens relative to the same subset from WT (Figure 2B). Consistent with the negative regulation of stem cell transcription factors in spleen by CD47-dependent thrombospondin-1 signaling,^17^ these results support prior evidence that that loss of *thbs1* or *cd47* results in accumulation of early hematopoietic precursors that are Ter119^−^ and demonstrates a *cd47*^−/−^ -specific enrichment of cKit^+^Ter119^+^ committed erythroid precursors. Proliferating cells with low Ki67 expression were more abundant in the Ter119^+^ and Ter119^−^ populations of *cd47*^−/−^ cells relative to WT and *thbs1*^−/−^cells, suggesting that loss of *cd47* but not *thbs1* increases the proliferation of committed erythroid precursors (Figure 2C, D).

### CD47 limits proliferation of Ter119^+^CD34^−^ and Ter119^−^CD34^+^ spleen cells

Although Figure 1 demonstrated increased numbers of Ter119^+^ and CD34^+^ cells in *cd47*^−/−^ spleens, further analysis revealed that more CD34^+^ cells are Ter119^−^ than Ter119^+^ (Figure 2E,G left panels). Therefore, loss of CD47 upregulates both early Ter119^−^CD34^+^ progenitors and more mature Ter119^+^ progenitors, most of which have lost CD34 expression (Figure 2F). Most of the increased proliferation of *cd47*^−/−^ spleen cells, indicated by Ki67 expression, was in the Ter119^+^CD34^−^ subset (Figure 2F, center and right panels), but proliferating *cd47*^−/−^ cells were also enriched in the Ter119^−^CD34^+^ population (Figure 2G). These data indicate roles for more differentiated Ter119^+^/CD34^−^ erythroid progenitors as well as earlier Ter119^−^/CD34^+^ erythroid progenitors in mediating the increased extramedullary erythropoiesis in *cd47*^−/−^ spleens.

### Identification of CD47-dependent erythroid precursor populations

**Figure 3:**
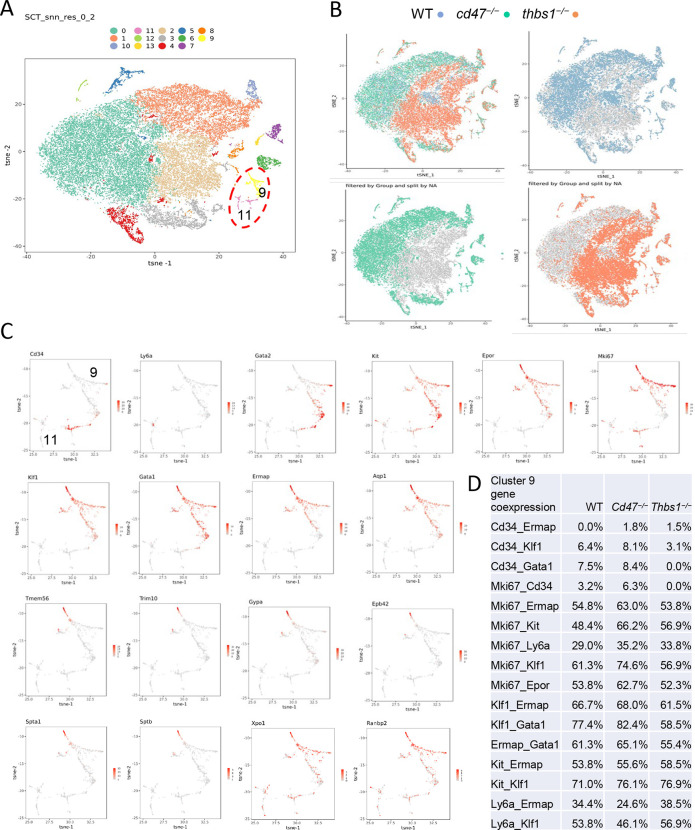
Effects of *cd47* and *thbs1* gene deletion on stem cell and erythroid precursor populations in mouse spleen identified using single cell RNA sequence analysis. (A) tSNE clustering analysis of lineage-depleted spleen cells from WT, *cd47*^−/−^ and *thbs1*^−/−^mice. The encircled area contains erythroid cells (clusters 9) and stem cells (cluster 11). (B) Distribution of WT, *cd47*^−/−^ and *thbs1*^−/−^ spleen cells in each cluster of the tSNE plot. (C) High resolution tSNE plots showing the distribution of the multipotent stem cell markers CD34 and Ly6a (Sca1) and Gata2, the erythropoietic markers Kit and Epor, the proliferation marker Mki67, erythroid differentiation transcription factors Klf1 and Gata1, and erythroid differentiation and extramedullary erythropoiesis markers Ermap, Aqp1, Tmem56, Trim10, Gypa1, Spta1, Sptb, Ebp42, Xpo1, and RanBP2 in clusters 9 and 11. (D) Co-expression of the indicated erythropoiesis related genes in WT, *cd47*^−/−^ and *thbs1*^−/−^ spleen cells in cluster 9 was quantified and expressed as a percentage of the total cell number of each genotype in cluster 9.

Single cell RNA sequencing (scRNAseq) was used to further define effects of CD47 and thrombospondin-1 on erythroid precursors in spleen (supplemental Figures 3,4). Spleen cells from WT, *cd47*^−/−^ and *thbs1*^−/−^ mice were treated with immobilized antibodies to deplete monocytic, T, B, and NK cell lineages and mature RBC and subjected to scRNAseq analysis. Following alignment, the mRNA expression data were clustered in two dimensions using the t-distributed stochastic neighbor embedding (tSNE) method in NIDAP. The spleen cells clustered in 13 groups (Figure 3A).

Cell type analysis using SingleR with Immgen and mouse RNAseq databases identified the main cell types in each cluster (supplemental Figure 4A). WT, *cd47*^−/−^ and *thbs1*^−/−^ cells clustered by genotype in the major residual T cell clusters 0, 1, and 2 but had similar distributions within clusters 9 and 11 (Figure 3B). Immgen main cell type annotation identified cluster 9 and a subset of cluster 11 as stem cells, and mouse RNA seq annotation identified erythrocyte signatures mostly in cluster 9. Erythroid-specific markers were found mainly in cluster 9 (Figure 3C and supplemental Figures 4B, 5). Consistent with the flow data in figures 1 and 2, *cd47*^−/−^ cells were more abundant in cluster 9 (64%), and *thbs1*^−/−^ cells were less abundant (15%) relative to WT cells (21%).

CD34 was expressed mostly in cluster 11 and to lower parts of cluster 9, whereas Ly6a (Sca1) was restricted to isolated cells in both clusters. Kit and Gata2, which are expressed by multipotent progenitors through CFU-E, were expressed in lower and middle areas of cluster 9 and to a limited degree in cluster 11 (Figure 3C). Epor, Gata1, Klf1, Ermap, Aqp1 and the proliferation marker Ki67 had similar distributions in cluster 9 (Figure 3C). Trim10 and the late erythroid markers Gypa, Tmem56, Epb42, Spta1, and Sptb were restricted to the upper regions of cluster 9 (Figure 3C). Therefore, erythroid differentiation within cluster 9 correlates with increasing TSNE-2 scores.

**Figure 4.**
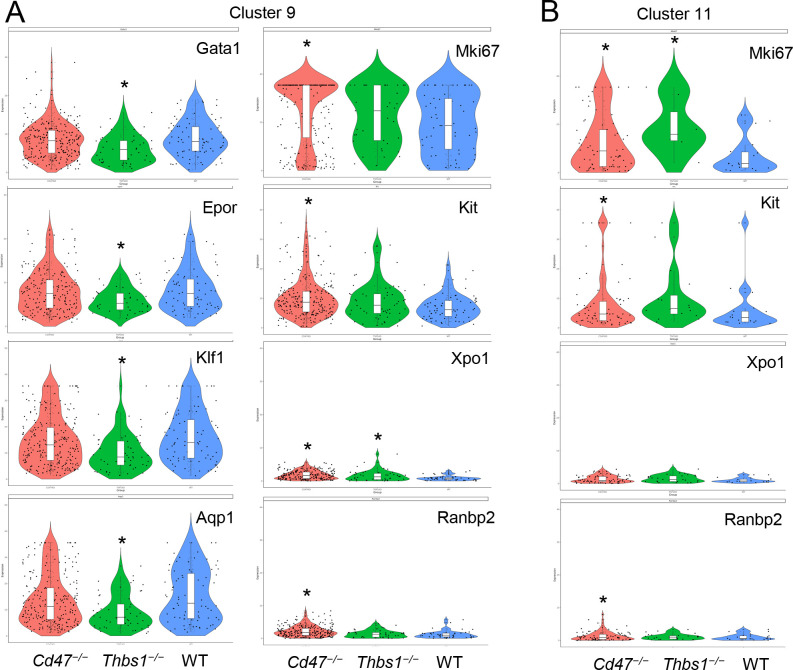
Differential effects of *cd47* and *thbs1* gene deletion on mRNA expression levels in cluster 9 erythroid precursor cells and proliferation of CD34^−^Ter119^+^Kit^+^ cells assessed by flow cytometry. (A) Violin plots comparing mRNA expression levels of the indicated genes in *cd47*^−/−^, *thbs1*^−/−^ and WT spleen cells in cluster 9. (B) Violin plots comparing mRNA expression levels of the indicated genes in *cd47*^−/−^, *thbs1*^−/−^ and WT spleen cells in cluster 11. * = p<0.05.

Similar high percentages of WT and *cd47*^−/−^ cells within cluster 9 expressed the erythroid lineage markers Klf1, Aqp1, Tfrc, Epor, Ermap, and Gata1, but percentages for some were lower in *thbs1*^−/−^ cells (supplemental Figure 6A). The average mRNA/cell for Tfrc and Ermap was significantly higher in *cd47*^−/−^ cells, whereas mRNAs encoding Klf1, Aqp1, Epor, and Gata1 were significantly lower compared to WT in *thbs1*^−/−^ cells (Figure 4A, Table 2). Violin plots indicated that cells with the highest Mki67 mRNA expression were more abundant in *cd47*^−/−^ cluster 9, and the average expression was higher (p=3.5×10^−5^), but cells with high and low Mki67 had similar distributions in *thbs1*^−/−^ and WT cells (Figure 4A). Mki67 expression levels were lower in cluster 11, and *cd47*^−/−^ cells had higher mean expression that WT, but and *thbs1*^−/−^ cells had lower Mki67 than WT (Table 2). Expression of Kit was also higher in *cd47*^−/−^ versus WT cells in cluster 9 (p=2.8×10^−4^). Although more *cd47*^−/−^ cells expressed Gata1 and Epor, the mean expression was not higher than in WT cells.

Expression of Xpo1 and Ranbp2, which regulates activity of the Xpo1/Ran complex that stabilizes Gata1,^33^ was significantly higher in cluster 9 than in cluster 11, and within cluster 9 the distribution of positive cells was similar to that for other markers of committed erythroid precursors (Figure 3C). The average expression in cluster 9 was significantly higher in *cd47*^−/−^ and *thbs1*^−/−^ cells compared to WT, whereas expression was less genotype-dependent in cluster 11 (Figure 4A,B, Table 2). Resolution of cluster 9 into CD34^+^ and CD34^−^ cells revealed that the CD47-dependent expression of Xpo1 and Ranbp2 was restricted to the CD34^−^ population (supplemental Figure 6B). In contrast, expression of mRNA for RanBP1, which regulates the physical interaction of Xpo1 with CD47,^5^ did not differ in *cd47*^−/−^ or *thbs1*^−/−^ cells in clusters 9 (Table 2). Nr3c1, a marker of adult definitive erythropoiesis,^25^ and Ddx46, which is required for hematopoietic stem cell differentiation,^34^ were among the genes with increased percentages of positive cells and significantly increased average expression in both *cd47*^−/−^ and *thbs1*^−/−^ cells in cluster 9 (supplemental Figure 6A, Table 2). These genes also showed CD47-dependent expression in cluster 11.

The co-expression of mRNAs for erythropoietic genes was compared in cluster 9 cells from *cd47*^−/−^, WT, and *thbs1*^−/−^ spleens (Figure 3D). Small fractions of the WT CD34^+^ cells expressed Gata1 and Klf1 mRNAs, 3.2% expressed Mki67, and none expressed Ermap. Consistent with the flow cytometry data in Figure 2, coexpression of the respective genes with CD34 was more frequent in *cd47*^−/−^ cells in cluster 9, but generally less in *thbs1*^−/−^ cells. Notably, CD34^+^ *thbs1*^−/−^ cells showed no Mki67 coexpression. In contrast, WT cells that expressed committed erythroid differential markers were highly proliferative. Coexpression of all these genes with Mik67 was more frequent in *cd47*^−/−^ cells compared to WT cells, but only for Kit and Ly6a in *thbs1*^−/−^ cells. Coexpression of the erythroid transcription factors Gata1 and Klf1 was similarly increased in *cd47*^−/−^ cells but decreased in *thbs1*^−/−^ cells compared to the WT cells in cluster 9. The latter is consistent with the extended life span of *thbs1*^−/−^ RBC.^9^ Kit coexpression with the erythroid lineage markers Ermap and Klf1 was moderately dependent on genotype, and Ly6a coexpression with these markers was decreased for *cd47*^−/−^ cells in cluster 9.

### Reclustering and analysis of cells expressing erythroid signature genes

**Figure 5.**
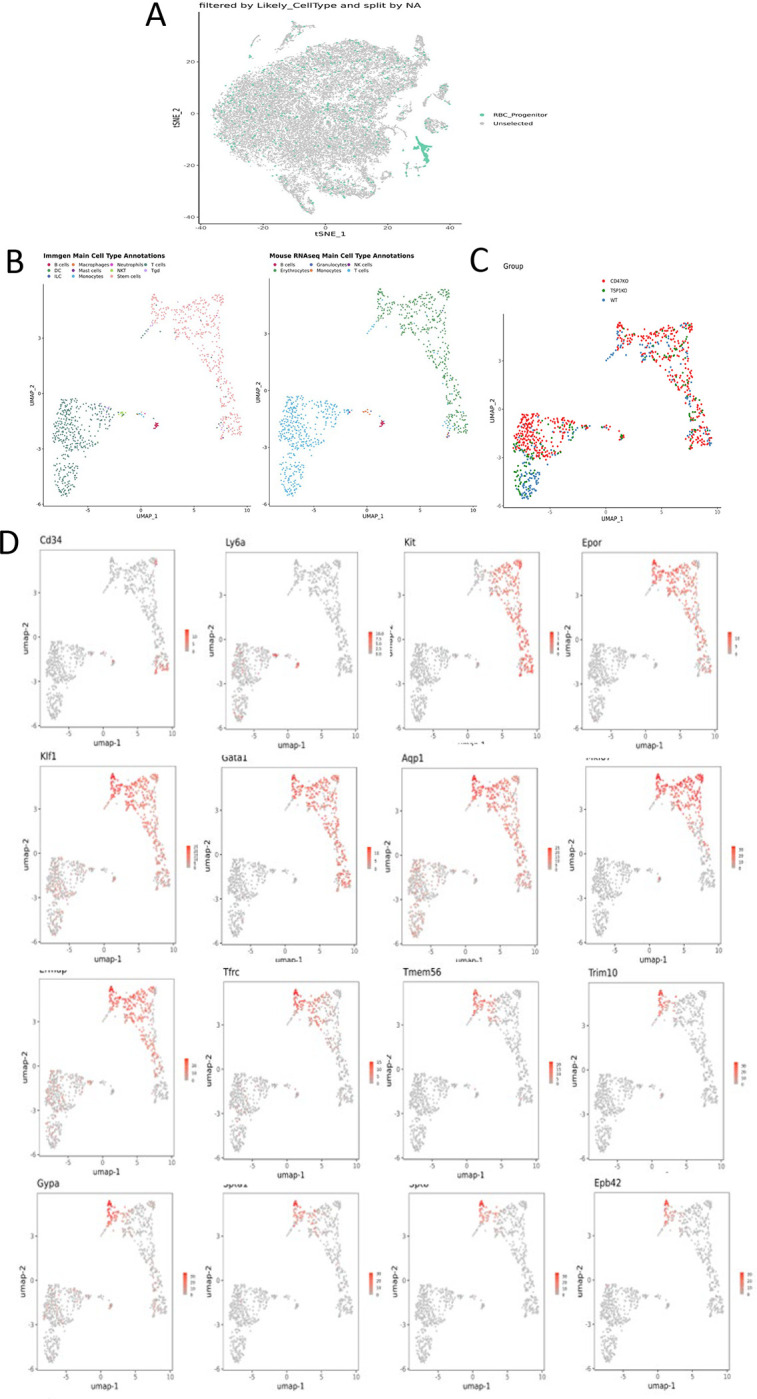
Re-clustering of lineage-depleted spleen cells selected for expression of erythroid signature genes. (A) tSNE plot showing the distribution of cells selected for expressing threshold levels of Gypa, Ermap, Klf1, and/or Aqp1. (B) Immgen and mouse RNAseq main cell type annotation of reclustered cells expressing the erythroid gene signature. Re-clustered cells are displayed in a UMAP projection. (C) Distribution of WT (blue), *cd47*^−/−^ (red), and *thbs1*^−/−^ cells (green) in the cluster UMAP projection. (D) Distribution of the multipotent stem cell markers CD34 and Ly6a (Sca1), erythropoietic markers Kit and Epor, the erythroid differentiation transcription factors Klf1 and Gata1, and erythroid differentiation and extramedullary erythropoiesis marker Aqp1, the proliferation marker Mik67, and the erythroid markers Ermap, Tfrc, Tmem56, Trim10, Gypa1, Spta1, Sptb, and Ebp42 in the T cell (lower left) and erythroid lineage cluster (upper right).

Erythrocyte progenitor markers were expressed mainly within cluster 9, but some positive cells were scattered across the T cell clusters 0, 1, and 2 (supplemental figures 4B, 5). To determine whether the latter positive cells include relevant erythroid lineages that were missed in the initial clustering, the lineage markers Gypa, Ermap, Klf1, and Aqp1 were selected as an erythroid signature to calculate module scores (Supplemental figure 7). Cells expressing this signature based on module scores (Figure 5A) were then reclustered, yielding two major clusters in a UMAP projection (Figure 5B). Immgen annotations predicted these represent T cells and stem cells (Figure 5B). Mouse RNAseq annotation confirmed the T cell cluster and predicted the stem cell cluster to be of erythrocyte lineage (Figure 5B). WT, *cd47*^−/−^ and *thbs1*^−/−^ cells were uniformly distributed throughout the erythroid cluster but segregated within the T cell cluster (Figure 5C). *Cd47*^−/−^ cells were more abundant in the erythroid cluster (310%) and *thbs1*^−/−^ cells were less abundant (70%) relative to WT cells.

A volcano plot indicated major differences in the transcriptomes of the two main clusters(Supplemental Figure 7B). Infrequent expression of Ermap, Klf1, and Aqp1 in the T cell cluster is consistent with previous reports of their expression in minor subsets of T cells^35–37^ (Figure 5D).

The distribution of erythroid markers throughout the reclustered erythroid population was consistent with the results for cluster 9 (Figure 5D). CD34^+^ cells were concentrated in the lower region of the cluster. Consistent with the selection of calls based on expression of committed erythroid lineage genes, the cluster lacked Ly6a^+^ cells. Kit was expressed by the CD34^+^ cells and extended upward through the cluster. The upper cells showed increased expression of Epor, Klf1, Gata1, and Aqp1. Expression of the proliferation marker Mki67 was strongest in the upper region of the cluster and extended to cells that cells that expressed markers of more mature precursors including Ermap, Tfrc (transferrin receptor), and Tmem56. Trim10 mediates terminal differentiation and colocalized with mRNAs encoding glycophorin A, spectrins A and B, band 4.2. The upregulation of erythrocyte lineage markers coincided with more abundant *cd47*^−/−^ cells in this cluster, which supports the initial clustering and confirms increased extramedullary erythropoiesis in *cd47*^−/−^ mice.

## Discussion

The properties of erythroid precursors that accumulate in *cd47*^−/−^ spleens are consistent with previous studies of stress induced extramedullary erythropoiesis associated with malaria or trypanosome infections.^23,24^ In addition to containing elevated NK precursors.^18^, the present data demonstrate that *cd47*^−/−^ spleens contain more abundant erythroid precursors that are Ter119^+^ by flow cytometry but presumably lack sufficient Ter119 for antibody bead depletion. These cells are present but less abundant in WT spleens, indicating that a low level of extramedullary erythropoiesis occurs in healthy mouse spleen. The Ter119^+^CD34^−^ cells that accumulate in *cd47*^−/−^ spleens are more proliferative and express multiple markers of committed erythroid precursors. In contrast, the same cells are depleted in *thbs1*^−/−^ spleen, consistent with the function of thrombospondin-1 to facilitate the CD47/SIRPα-mediated turnover of aging RBC. These data also indicate that physiological levels of thrombospondin-1 support a basal level of erythropoiesis in WT spleen.

Notably, *thbs1*^−/−^ and *cd47*^−/−^ spleens both contain more early erythroid precursors than are maintained basally in a WT spleen, consistent with the role of thrombospondin-1 signaling via CD47 to limit the expression of multipotent stem cell transcription factors in spleen.^17^ Earlier erythroid precursors that are Ter119^−^Kit^+^ accumulate in *thbs1*^−/−^ spleens. *Thbs1*^−/−^ and *cd47*^−/−^ cells express more Ddx46, which is required for differentiation of hematopoietic stem cells,^34^ and Xpo1, which supports the erythropoietic function of Gata1.^28^ Although Ter119^+^ cells expressing markers of committed erythroid progenitors were depleted in *thbs1*^−/−^ compared to WT spleens, s. mRNAs for some markers of committed erythroid cells including Nr3c1 mRNA were elevated *thbs1*^−/−^ and *cd47*^−/−^ in cluster 9 cells. However, early progenitors express CD45R, and inclusion of this antibody in the negative selection cocktail should deplete early progenitors from the populations used for both RNAseq analyses. This may account for the lack of CD47-dependent CD34^+^ cells in cluster 9.

One caveat in interpreting the CD47- and thrombospondin-1-dependence of the extramedullary erythropoiesis markers Ermap and Aqp1 in total spleen cells and the Ter119-depleted cells used for scRNAseq is that both are also expressed in minor subsets of T cells.^37^ The reclustering analysis confirmed that these erythropoietic markers are expressed in a minor T cell population, which notably also showed CD47-dependent gene expression changes. This may also account for the differences in CD47-dependence of erythropoietic marker expression observed by flow cytometry in Ter119^+^ cells but not in total spleen cells or Ter119^−^ spleen cells. In addition to assessing extramedullary erythropoiesis, the CD47-dependent erythropoiesis genes identified here may have translational utility. Therapeutics designed to inhibit function of CD47 have entered clinical trials for treating cancer, but anemia associated with loss of inhibitory SIRPα signaling in macrophages has been a frequent side effect for the first generation of these therapeutics.^38^ In addition to their accumulation in spleens of *cd47*^−/−^ mice, erythroid precursors may also appear in circulation, suggesting that some of the CD47-dependent erythroid genes identified in this study may be useful biomarkers for assessing side effects of CD47-targeted therapeutics.

## Materials and Methods

### Mice and cells

WT, *cd47*^−/−^ (B6.129S7-Cd47tm1Fpl/J), and *thbs1*^−/−^ (B6.129S2-*Thbs1*^*tm1Hyn*^/J) mice were obtained from The Jackson Laboratory, backcrossed on the C57BL/6 background, and maintained under specific pathogen free conditions. All animal experiments were carried out in strict accordance with the Recommendations for the Care and Use of Laboratory Animals of the National Institutes of Health under a protocol approved by the NCI Animal Care and Use Committee. Age matched 8–12 week old mice were used for experiments except where noted.

Spleens were removed from the mice and homogenized in HBSS and passed through a 70 μm mesh (Sigma, CLS431751) to remove debris. The cell suspension was treated with ACK lysis buffer for 4 minutes to lyse the RBC. The suspensions were centrifuged and washed twice with cold HBSS. Aliquots of single cell suspensions were stained using trypan blue and counted to assess viability.

### Flow cytometry

Single-cell suspensions from spleens were stained by incubation for 30 min at 4° C using optimized concentrations of antibodies: CD34-APC and Ter119-APC, cKit-PE and PE/Cy7, Sca1-PE/Cy7, Ki67-Percp/Cy5.5 and AlexaFl647 (Biolegend). Non-tagged Ermap, Gypa, Aqp1 (Thermofisher) and Epor (Bios Inc.) antibodies were detected using secondary goat anti-rabbit AF594 antibodies. Stained single cell suspensions were acquired on an LSRFortessa SORP (BD Biosciences), and data were analyzed using FlowJo software (Tree Star). A total of 2×10^5^ gated live events were acquired for each analysis. Isotype and unstained controls were used to gate the desired positive populations.

### Single cell RNA sequencing (scRNAseq)

Single cell suspensions from WT, *thbs1*^−/−^ and *cd47*^−/−^ spleens were depleted of all mature hematopoietic cell lineages including erythroblasts and mature RBC by passing through CD8a (Ly-2) microbeads and CD8a+ T Cell Isolation Kit, mouse (Miltenyi Biotec). Single cell suspensions were incubated with the supplied antibody cocktail of the CD8+ T cell isolation kit for 15 min. on ice and then passed through the column as per manufacturer’s instructions. The flow through combined with 3 washes was centrifuged, and the cells were incubated with the CD8a (Ly-2) microbeads and then passed through magnetic columns to obtain lineage-depleted cell populations. Capture & Library Preparation for single cell end-counting gene expression using the 10X Genomics platform was performed by the Single Cell Analysis Facility (CCR). Data analysis was performed using the Seurat workflow (v.3.1.5).^39^ Cell types were called using SingleR (v.1.0) ^40^ and Immgen and Mouse RNAseq databases.

#### Capture & Library Preparation –

The cell samples received two washes in 1 mL of ice-cold PBS to remove any ambient contaminating mRNA from the suspension. The sample volume was reduced to ~100 μL, and the sample concentration and viability were assessed using the LunaFL fluorescent cell counter. the viability was more than 90% for all the samples. Samples were loaded according to the 10× 3’ v3 User guide with a single capture lane per sample targeting a recovery of 6,000 cells per lane. Cell partitioning completed successfully with uniform emulsion consistency and the reverse transcription PCR was run overnight. All subsequent steps of library preparation and Quality Control were performed as described in the 10X Genomics 3’ v3 Single Cell User Guide. After the RT step, the emulsion was broken, and amplification of the 10× barcoded cDNA was completed. Amplified cDNA was then used for the construction of 3′ gene expression libraries. Each cDNA library was sequenced on a NovaSeq platform (Illumina) to generate 150-bp paired-end reads.

#### Data Analysis –

We set filters to exclude genes found in fewer than 3 cells, cells with fewer than 200 genes, cells with fewer than 500 UMI, cells with complexity (genes/UMI) lower than 0.5, and cells exceeding 3 median absolute deviations for both mitochondrial expression percentage and the number of genes per cell. The dimensionality reduction plots were based on 22 principal components. We chose a clustering resolution of 0.2 and found two clusters (9 and 11, Figures 3) to contain most of the RBC progenitor cells this dataset. Downstream analysis and visualization were performed within the NIH Integrated Analysis Platform (NIDAP) using R programs developed on the Foundry platform (Palantir Technologies) (Supplemental Figures 2 through 4, 6). Additionally, RBC progenitor markers (Gypa, Ermap, Klf1, and Aqp1) were used to construct a module score by which likely RBC progenitor cells were identified using a manually set threshold, filtered, and further analyzed by filtering (Figure 4) and reclustering (Figure 5) to include only those cells (Supplemental Figure 7).

### Bulk RNAseq

#### Sample preparation from mouse spleens –

Cells were isolated from spleens of 4–6-week-old WT and *cd47*^−/−^ C57Bl/6J mice (n=4). Cell isolation buffer contained phosphate-buffered saline (PBS), pH 7.2, 0.5% bovine serum albumin (BSA), and 2 mM EDTA and kept on ice. The buffer was filtered using Corning^®^ 50 mL Tube Top Vacuum Filter System, 0.22 μm Pore 13.6cm^2^ CA Membrane, Sterile (Corning). Dissected spleens were immediately placed in Petri dishes containing PBS (Gibco), and homogenized by pressing spleens through a 70 μm cell strainer using the back of plastic transfer pipettes (Sigma) with 5 ml of buffer. Mature RBC were lysed using 1 ml of ACK lysis buffer (Quality Biologicals) at room temperature for 5 minutes according to the manufacturer’s instructions.

#### Isolation and RNAseq analysis of lineage depleted populations containing CD8^+^ T cells –

Naïve CD8-enriched lineage-depleted spleen cells were prepared using the CD8a+ T Cell Isolation Kit. Clumps of cells were removed using 30 μm Pre-Separation Filters. The suspension was subjected to negative selection of CD8^+^ T cells by incubating with biotin-conjugated monoclonal antibodies against CD4, CD11b, CD11c, CD19, CD45R (B220), CD49b (DX5), CD105, MHC Class II, Ter-119, and TCRγ/δ and passing through columns to deplete the indicated lineages according to manufacturer’s instructions (Miltenyi Biotec 130–104-075). The freshly isolated CD8^+^ T cells plus unselected lineages were washed with filtered phosphate-buffered saline (PBS), pH 7.2, containing 0.5% bovine serum albumin (BSA), and 2 mM EDTA. The cells were immediately placed at −80 °C until the RNA was extracted, subjected to RNAseq analysis, and analyzed for differential gene expression using the NIDAP pipeline as described.^41^ Preranked gene set enrichment analysis of the Hallmark collection was performed using the t-statistic as ranking variable (Supplemental Figure 1). This analysis was also performed using R programs run on NIDAP.

## Supplementary Material

Supplement 1

## Figures and Tables

**Table 1.** Expression of erythropoiesis-associated genes in lineage-depleted *cd47^−/−^* versus WT spleen cells

Gene	Erythropoiesis expression/function	Fold change *cd47^−/−^/WT*^a^	T Statistic	P-Value

***Ermap***	extramedullary erythropoiesis marker^b^	21.5	6.04	9.35x10^−5^
***Tal1***	extramedullary erythropoiesis marker^b^	13.9	5.13	3.55x10^−4^
***Gypa***	extramedullary erythropoiesis marker^b^	89.3	3.67	3.86x10^−3^
***Gata1***	extramedullary erythropoiesis marker^b^	7.69	4.86	5.38x10^−4^
***Kel***	extramedullary erythropoiesis marker^b^	29.0	4.61	8.04x10^−4^
***Slc4a1***	extramedullary erythropoiesis marker^b^	151.4	4.90	5.05x10^−4^
***Klf1***	extramedullary erythropoiesis marker^b^	20.6	5.06	3.95x10^−4^
***Cldn13***	extramedullary erythropoiesis marker^b^	49.2	4.42	1.09 x10^−3^
***Trim10***	extramedullary erythropoiesis marker^b^	49.7	4.11	1.83 x10^−3^
***Epor***	extramedullary erythropoiesis marker^b^	12.3	4.91	5.01x10^−4^
***Sptb***	extramedullary erythropoiesis marker^b^	36.5	5.21	3.16x10^−4^
***Rhag***	extramedullary erythropoiesis marker^b^	33.0	4.64	7.68x10^−4^
***Hba-a1***	erythroblasts	63.8	6.46	5.23x10^−5^
***Hbb-bs***	erythroblasts	59.2	6.60	4.34x10^−5^
***Gata1***	BFU-E through erythroblasts	7.69	4.86	5.38x10^−4^
***Tfrc* (CD71)**	CFU-E through erythroblasts	3.12	6.19	7.58x10^−5^
***Kit***	Progenitors through CFU-E	1.72	6.24	7.02x10^−5^
***Sox6***	Adult definitive erythropoiesis	35.5	3.56	0.0046
***Aqp1***	Adult definitive erythropoiesis	26.9	6.51	4.91x10^−5^
***Nr3c1***	Adult definitive erythropoiesis	1.12	2.80	0.018
***Mki67***	Proliferation marker	4.43	7.65	1.14x10^−5^
***Cd34***	Multipotent progenitors through CFU-E	1.60	1.59	0.141
***Ly6a* (Sca1)**	Multipotent progenitors	1.17	1.02	0.331
***Anpep* (CD13)**	Multipotent progenitors	1.16	1.14	0.28
***Cd33***	Multipotent progenitors	-1.06	-0.37	0.71
***Gata2***	Multipotent progenitors	1.08	0.66	0.52
***Xpo1***	Stability of nuclear Gata1	1.23	3.86	2.7x10^−3^

a Gene enrichment in naïve *cd47^−/−^* vs WT spleen cells depleted for CD4, CD11b, CD11c, CD19, CD45R (B220), CD49b (DX5), CD105, MHC Class II, Ter-119, and TCRγ/δ..

b Reported markers of stress-induced extramedullary erythropoiesis ^23,24^

**Table 2.** Differential mRNA expression of erythropoietic, stem cell, and proliferation associated markers in WT, *cd47^−/−^*, and *thbs1^−/−^* cells in clusters 9 and 11.

Cluster	Gene	*cd47^−/−^* vs WT		*thbs1^−/−^* vs WT	

		p-value	Avg log_2_ FC	p-value	Avg log_2_ FC

**9**	Klf1	0.485	−0.087	0.0015	−0.374
**9**	Aqp1	0.52l	−0.084	0.0024	−0.338
**9**	Tfrc	0.0099	0.380	0.99	0.013
**9**	Epor	0.577	−0.041	0.0094	−0.198
**9**	Ermap	8.81x10^−4^	0.234	0.78	−0.053
**9**	Gata1	0.63	0.003	9.35x10^−4^	−0.267
**9**	Mki67	3.54x10^−5^	0.703	0.44	0.302
**11**	Mki67	0.0048	0.575	0.0031	−0.022
**9**	Kit	2.85 x10–4	0.253	0.092	0.174
**11**	Kit	0.038	0.267	0.71	0.127
**9**	Xpo1	3.95x10^−8^	0.317	0.0069	0.227
**11**	Xpo1	0.085	0.147	0.40	0.092
**9**	Ranbp1	0.20	0.075	0.11	0.048
**11**	Ranbp1	0.87	0.064	0.015	−0.351
**9**	Ranbp2	4.34x10^−15^	0.537	0.074	0.188
**11**	Ranbp2	0.0044	0.266	0.92	0.032
**9**	Nr3c1	3.82x10–4	0.246	8.85x10^−4^	0.302
**11**	Nr3c1	0.0075	0.110	1.37x10^−4^	0.303
**9**	Ddx46	2.87x10–8	0.382	1.88x10^−9^	0.518
**11**	Ddx46	5.61x10–4	0.292	0.0025	0.352

## Data Availability

Data supporting this publication have been deposited in NCBI’s Gene Expression Omnibus and are accessible through GEO Series accessions GSE239430. Reviewers may access this embargoed data by using the following Reviewer Token: qzuzwawmhpenxel. The code used to produce bioinformatics results can be found at https://github.com/NIDAP-Community/Regulation-of-Extramedullary-Erythropoiesis-by-CD47-and-THBS1. Additional data may be found in a data supplement available with the online version of this article.
