# Supplementary material for: Differential regulation by CD47 and thrombospondin-1 of extramedullary erythropoiesis in mouse spleen
Source: bioRxiv. 2024 Apr 4:2023.09.28.559992. Originally published 2023 Sep 28. Preprint. [Version 2] doi: 10.1101/2023.09.28.559992 (PMC10557659; doi:10.1101/2023.09.28.559992)
Supplement: Supplement 1 [file NIHPP2023.09.28.559992v2-supplement-1.pdf]

bioRxiv preprint doi: https://doi.org/10.1101/2023.09.28.559992; this version posted April 4, 2024. The copyright holder for this preprint (which was not certified by peer review) is the author/funder. This article is a US Government work. It is not subject to copyright under 17 USC 105 and is also made available for use under a CC0 license.

Figure Supplement Legends

Figure 1−figure supplement 1. Enlargement of spleen in the absence of CD47 and bulk RNAseq analysis of lin− *cd47*$^{-/-}$ vs WT spleen cells. A) Representative images of spleens from WT, *cd47*$^{-/-}$ and *thbs1*$^{-/-}$ mice. B) volcano plot for differentially expressed genes between lineage-depleted Naïve *cd47*$^{-/-}$ vs WT CD3^{+} spleen cells. Red dots represent significant genes with >2-fold change and p<0.001, and the top 30 genes were labelled. C). GSEA plot showing RBC Heme Metabolism gene set enrichment and, D) Heat map visualization of the top differentially expressed genes in the RBC Heme Metabolism gene set.

Figure 1−figure supplement 2. Flow cytometry analysis strategy. The sequential flow cytometry gating strategy is illustrated.

Figure 2−figure supplement 1. Flow cytometry analysis strategy. The sequential flow cytometry gating strategy is illustrated.

Figure 3−figure supplement. Single cell RNA sequence post-filter QC plots. The scatter plots, histograms and violin plots of three samples lineage-depleted of WT, *cd47*$^{-/-}$ and *thbs1*$^{-/-}$ spleen cells are shown. The number of UMI per cell filter was set to exclude cells with < 2000 UMI in nCount RNA. The Feature RNA plot shows the number of genes with non-zero expression detected per cell. Filtering was set to exclude cells having <15% mitochondrial gene expression as presented in the percent mt plot. The log10Genes per UMI plot represents the scores for complexity of the RNA library found in each cell. No filter was set in this row.

Figure 4−figure supplement 1. The distributions of mRNA expression of the indicated genes related to stem cells and erythropoietic cells are shown throughout the 18 clusters as a tSNE projection.

Figure 4−figure supplement 2. Violin plots of erythropoietic, stem cell, and proliferation associated marker mRNAs expressed in 18 cell clusters.

Figure 4−figure supplement 3. Differential expression of erythropoietic, stem cell, and proliferation associated markers in cell clusters 12 and 14. (A) Percentages of cells with detectable mRNA expression of the indicated erythropoietic, stem and proliferative markers in cluster 12 and cluster 14 in WT, cd47$^{-/-}$, and *thbs1*$^{-/-}$ spleens. (B) Differential mRNA expression of the nuclear export protein Xpo1 and nuclear pore protein synthesis instructor Ranbp2, which increase erythropoiesis by stabilizing Gata1 in the nucleus, in cluster12 and CD34+ and CD34− subsets of cluster 12 cells. (C) Differential expression of mitochondrial-encoded genes in cluster 12 cells from WT, cd47$^{-/-}$, and *thbs1*$^{-/-}$ spleens.

Figure 6−figure supplement 1. Strategy for reclustering spleen cells that express a gene signature for committed erythroid precursors. (A) The left panels show the distribution of cells expressing threshold levels of the 5 gene signature in the original TSNE projection. (B) The

bioRxiv preprint doi: https://doi.org/10.1101/2023.09.28.559992; this version posted April 4, 2024. The copyright holder for this preprint (which was not certified by peer review) is the author/funder. This article is a US Government work. It is not subject to copyright under 17 USC 105 and is also made available for use under a CC0 license.

RBC progenitor module scoring was performed, as module Scores (a.k.a. Signature Scores) calculated for each cell using expression of five genes (Gypa, Ermap, Klf1, Gata1, and Aqp1). The threshold was set manually (red dashed line on subplots) to separate high-scoring cells from low-scoring cells and expressed by the color on the TSNE plot (top left, high score cells are dark red and low scores cells are represented as light red). RBC Progenitor Module Scoring Plots show the distributions of module scores in lineage-depleted spleen cells from each mouse, with the threshold score indicated by the dotted red lines. (C) The volcano plot presents differentially expressed genes contrasting the reclustered erythropoietic progenitor cell clusters and the T cell cluster.

Figure 7−figure supplement 1. Differential expression of erythropoietic, stem cell, and proliferation associated markers in reclustered erythroid and T cell clusters. The percent of cells positive for expression of the indicated genes in WT, cd47$^{-/-}$, and *thbs1*$^{-/-}$ cells is presented in the indicated clusters. (-) indicates no cells in the cluster expressed detectable levels of the indicated gene.

Figure 1−figure supplement 1. Enlargement of spleen in the absence of CD47 (A) and bulk RNAseq analysis of lin⁻ *cd47*$^{-/-}$ vs WT spleen cells (B-D)

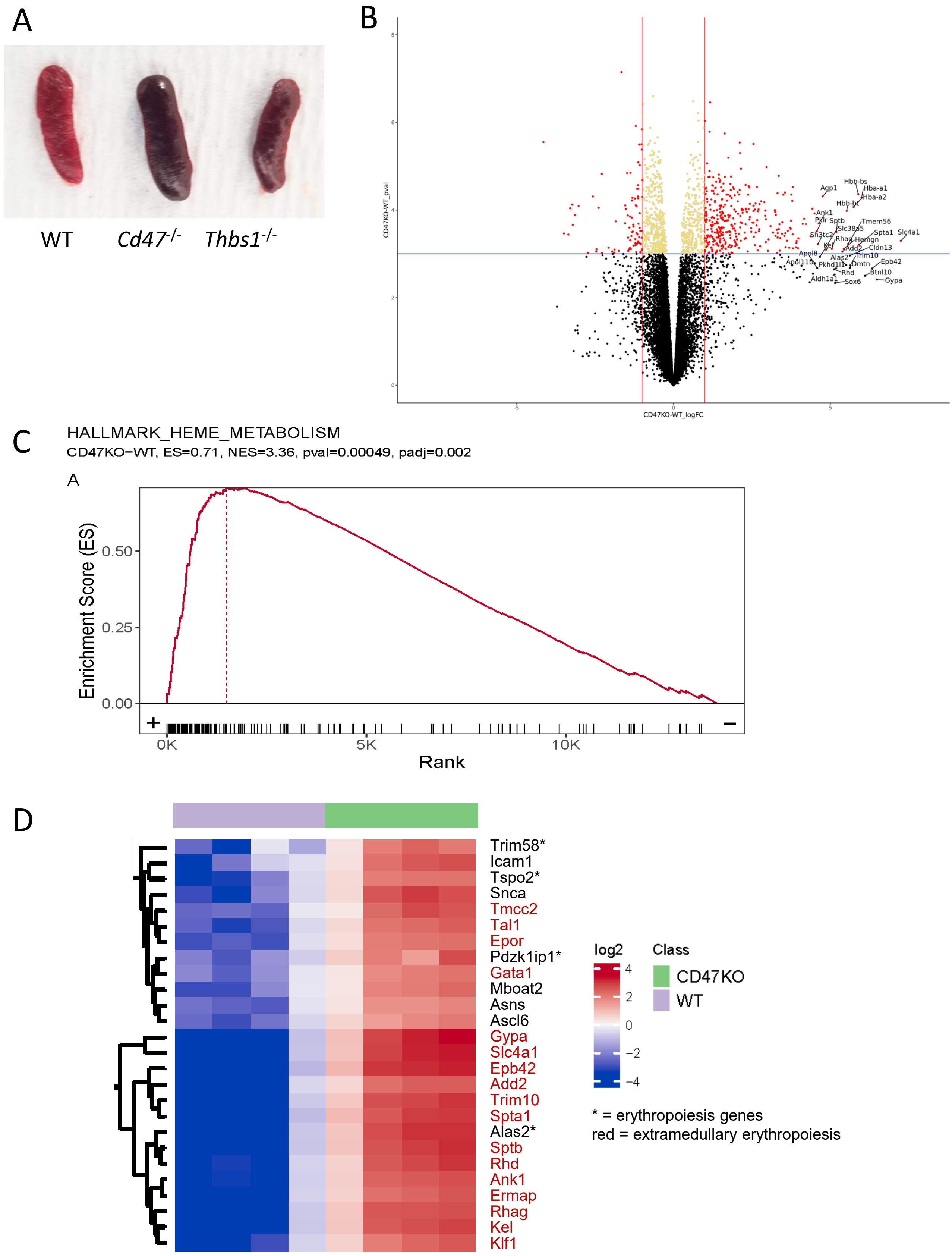

Figure 1−figure supplement 2. Flow cytometry diagram

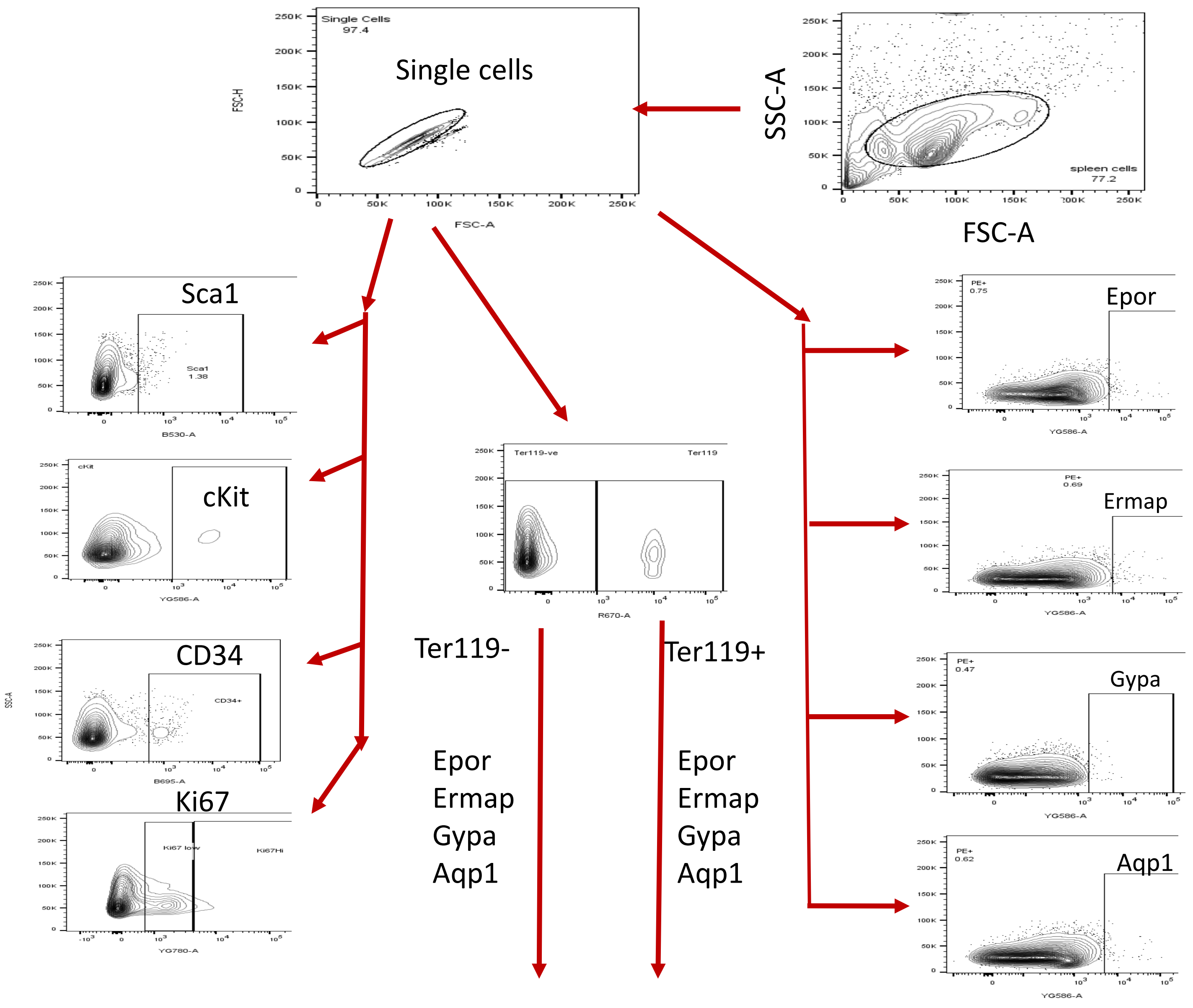

Figure 2−figure supplement 1. Flow cytometry gating

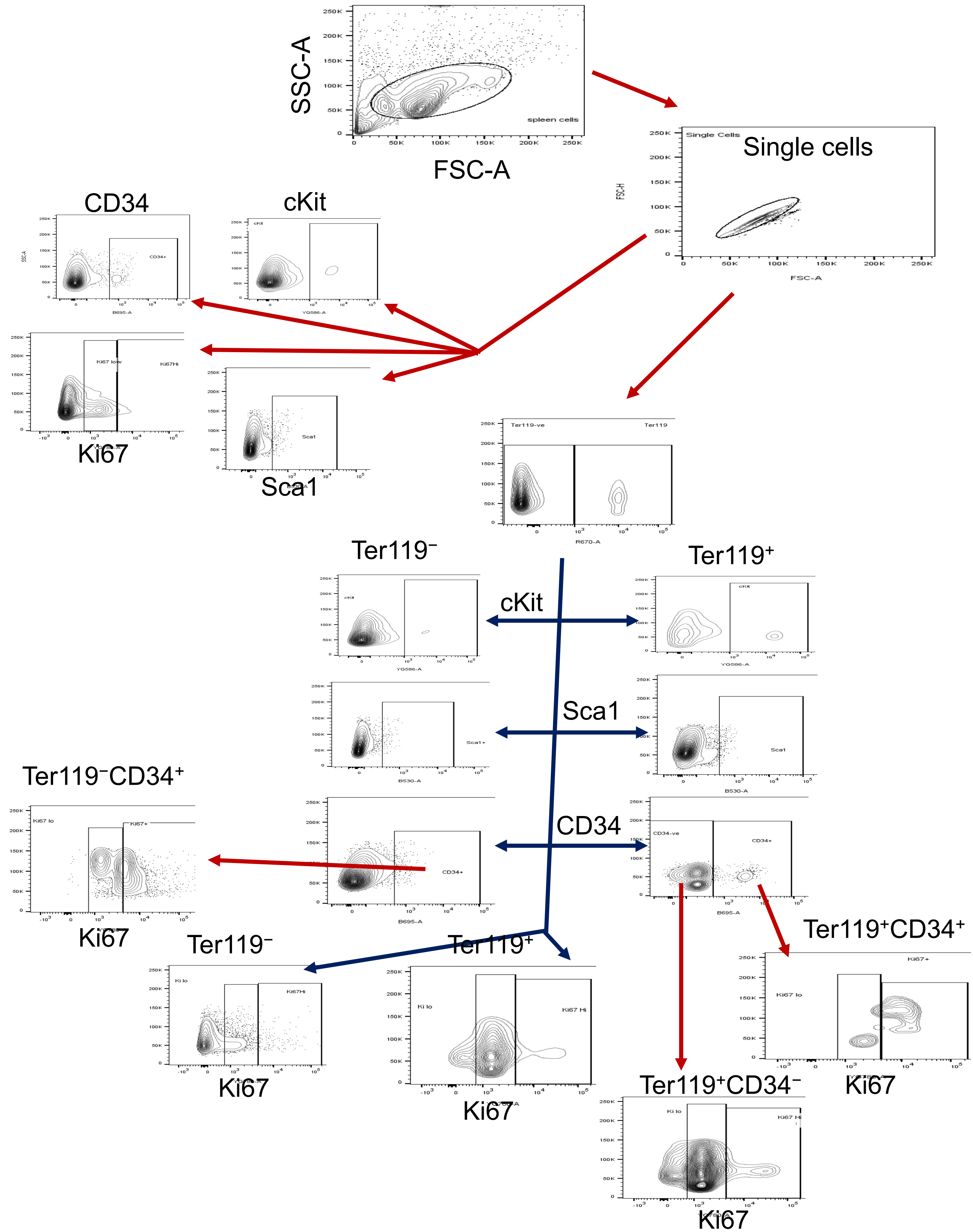

Figure 3−figure supplement 1 scRNAseq Post-Filter QC Plots

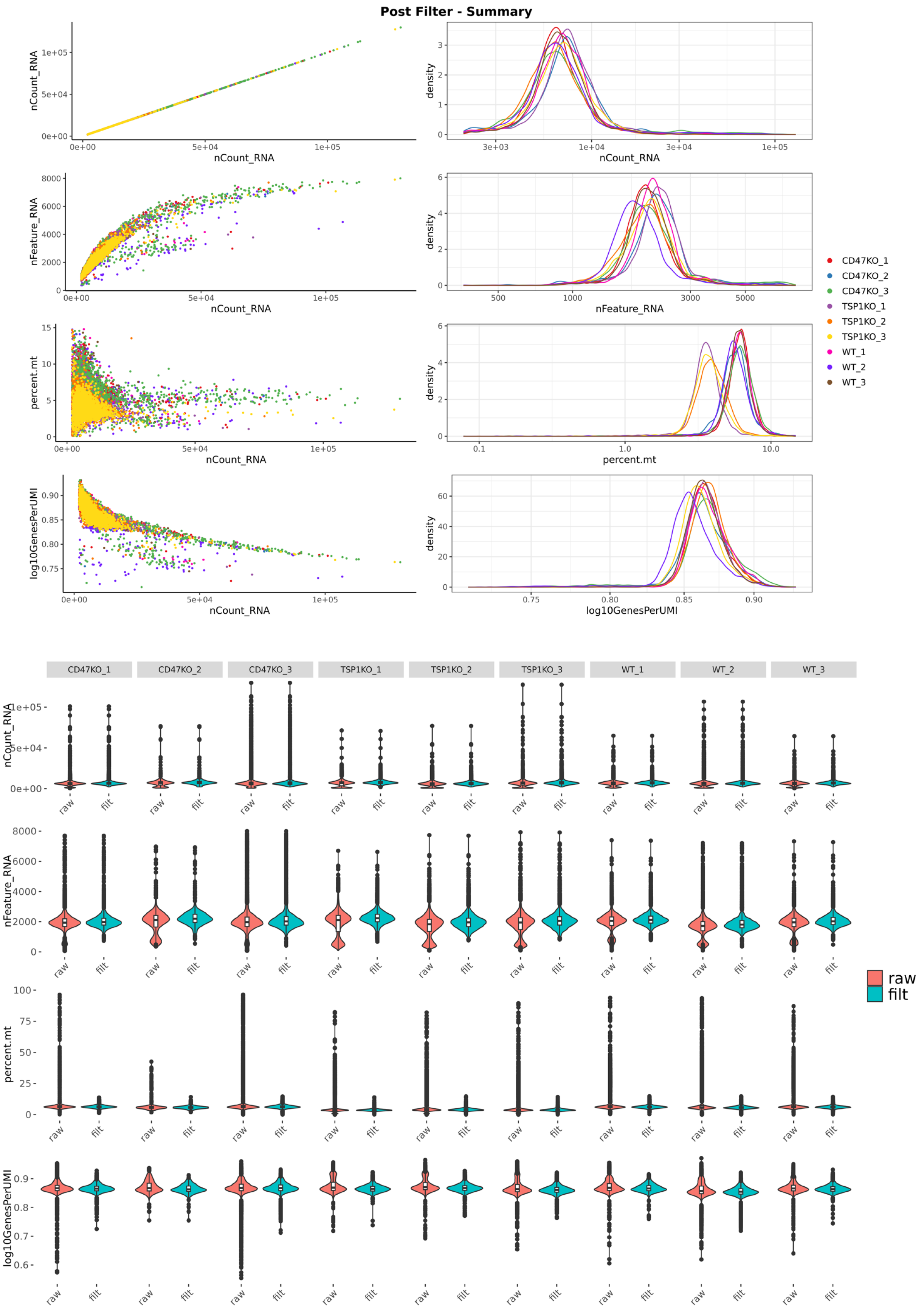

Figure 4−figure supplement 1. mRNA expression of the indicated genes.

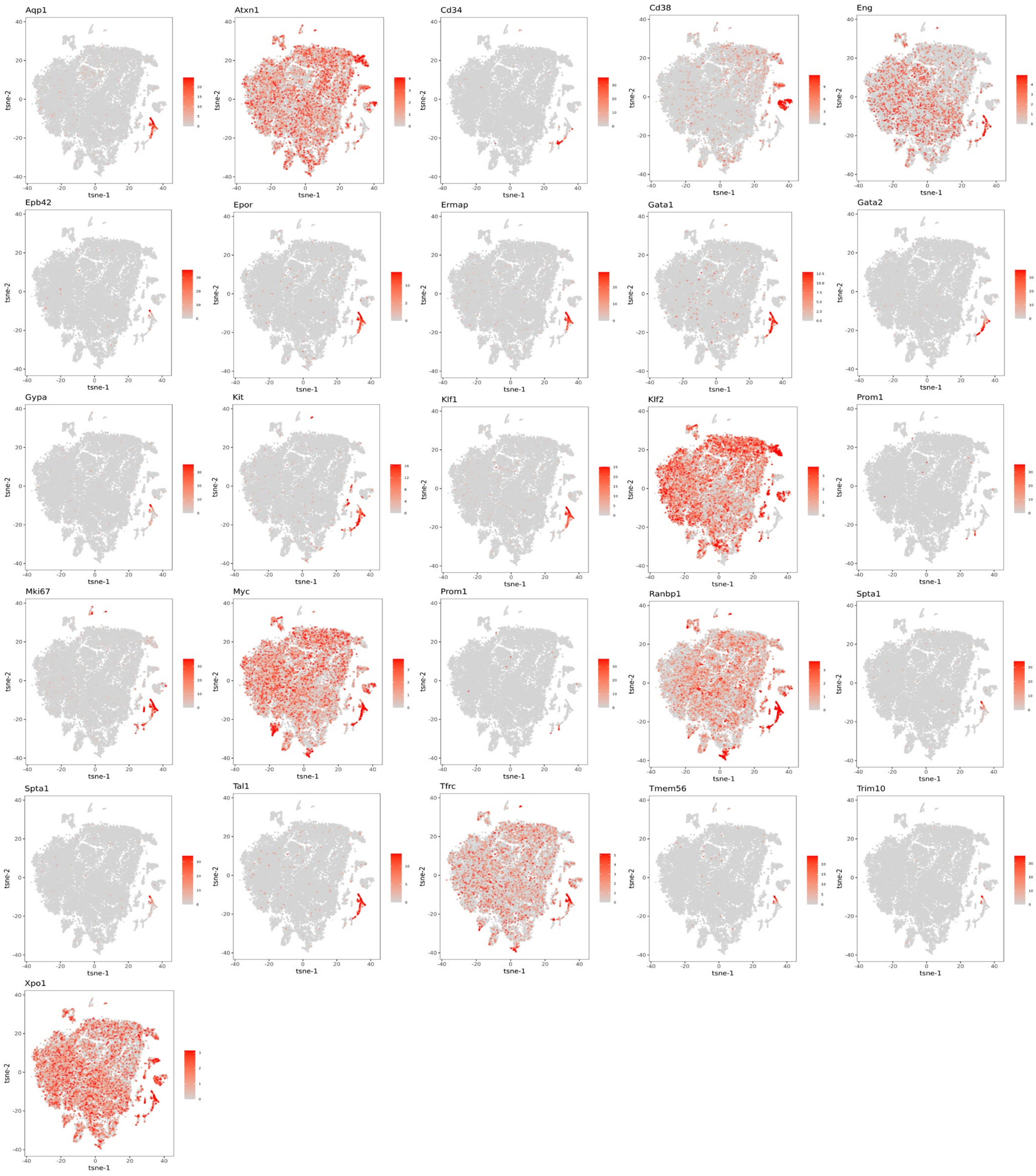

Figure 4−figure supplement 2. Violin plots of erythropoietic, stem cell, and proliferation associated markers in 18 cell clusters

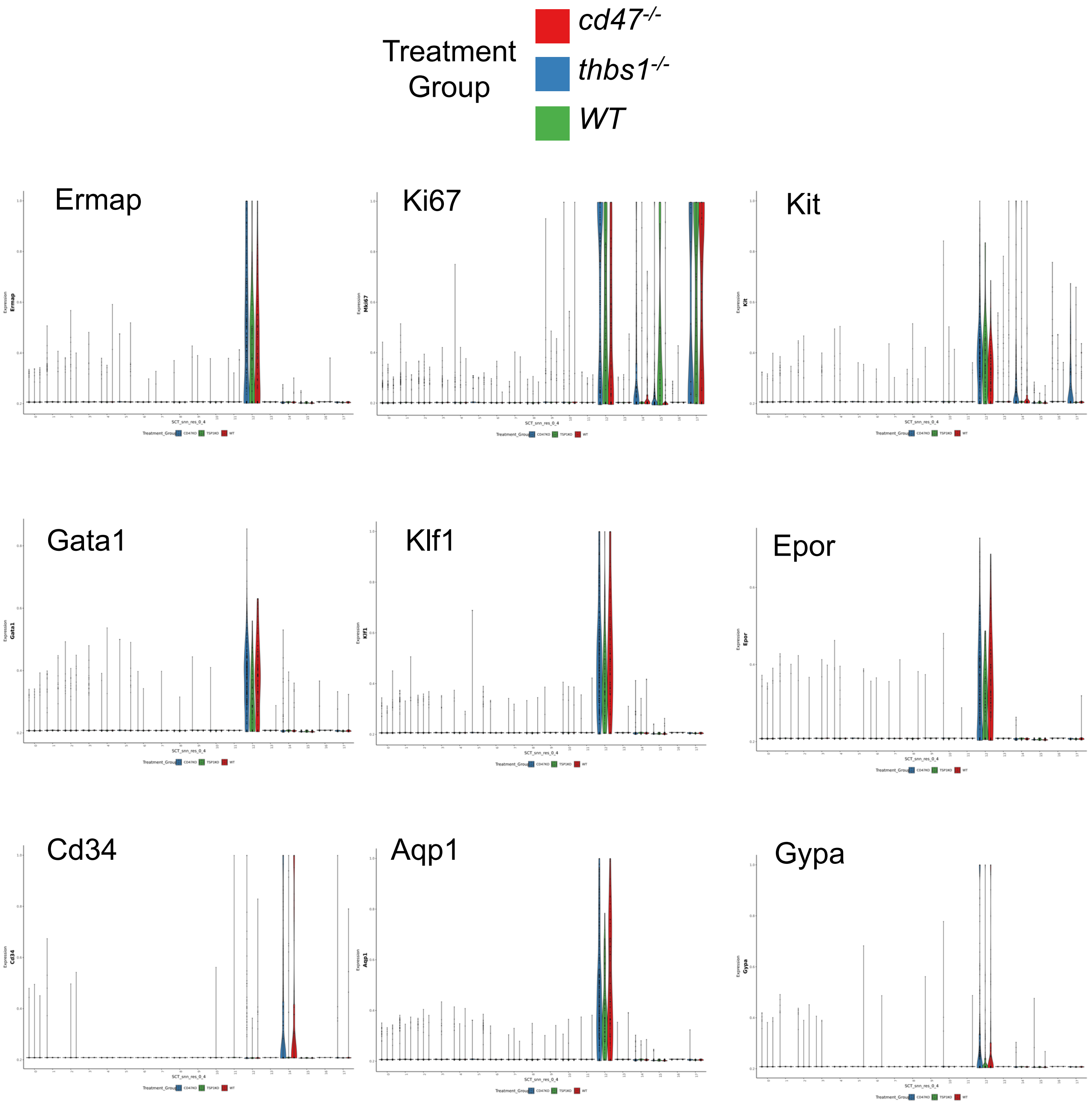

Figure 4−figure supplement 3. Differential expression of erythropoietic, stem cell, and proliferation associated markers in cell clusters 12 and 14 (A,B) and mitochondrial encoded genes in cluster 12 (C).

A

Cluster	Gene	% positive cd47$^{-/-}$ cells	% positive thbs1$^{-/-}$ cells	% positive WT cells
12	Klf1	88.0	79.7	89.1
14	Klf1	-	-	-
12	Aqp1	80.3	78.1	80.4
14	Aqp1	-	-	-
12	Tfrc	44	34.4	35.9
14	Tfrc	13.5	10.7	9.1
12	Epor	58.5	51.6	65.2
12	Ermap	66.9	65.6	64.1
12	Gata1	77.1	53.1	73.9
12	Mki67	74.6	64.1	57.6
14	Mki67	41.1	9.3	24.2
12	Kit	80.6	75.0	67.4
14	Kit	30.7	20.0	13.6
12	Xpo1	67.6	53.1	38.0
14	Xpo1	38.0	30.7	25.8
12	Ranbp1	95.8	98.4	91.3
14	Ranbp1	71.2	54.7	69.7
12	Ranbp2	85.9	64.1	47.8
14	Ranbp2	57.7	38.7	34.8
12	Nr3c1	63.4	68.8	42.4
14	Nr3c1	47.2	64.0	28.8
12	Ddx46	78.9	87.5	50.0
14	Ddx46	50.9	50.7	25.8

B

Cell cluster	mRNA	Cd47$^{-/-}$ vs WT		Thbs1$^{-/-}$ vs WT	
		p-value	Avg FC	p-value	Avg FC
C12	Xpo1	3.3×10^{-8}	1.39	0.0069	1.25
C12 (CD34^{-})	Xpo1	2.3×10^{-8}	1.42	0.010	1.25
C12 (CD34^{+})	Xpo1	NS		-*	
C14	Xpo1	NS		NS	
C12	Ranbp2	4.3×10^{-14}	1.69	NS	
C12 (CD34^{-})	Ranbp2	3.8×10^{-15}	1.76	NS	
C12 (CD34^{+})	Ranbp2	NS		-*	
C14	Ranbp2	0.0055	1.29	NS	

*Insufficient CD34^{+} *thbs1*$^{-/-}$ cells to calculate

C

Gene	P-val *Cd47*$^{-/-}$ vs WT	Avg log2FC *Cd47*$^{-/-}$ vs WT	P-val *Thbs1-/-* vs WT	Avg log2FC *Thbs1-/-* vs WT
mt-Atp6	0.887	-0.027	5.43×10^{-11}	-0.812
mt-Atp8	7.23×10^{-20}	0.98	0.0168	0.377
mt-Co1	0.028	0.135	2.60×10^{-7}	-0.532
mt-Co2	0.226	0.068	3.37×10^{-10}	-0.693
mt-Co3	0.207	0.097	1.02×10^{-8}	-0.671
mt-Cytb	0.338	-0.006	9.35×10^{-10}	-0.790
mt-Nd1	0.074	0.15	6.78×10^{-6}	-0.636
mt-Nd2	0.135	0.113	1.00×10^{-7}	-0.748
mt-Nd3	7.67×10^{-9}	0.531	1.36×10^{-7}	-0.813
mt-Nd4	0.152	0.093	5.84×10^{-6}	-0.558
mt-Nd4l	3.50×10^{-10}	0.658	6.49×10^{-8}	0.787
mt-Nd5	4.63×10^{-8}	0.612	0.0154	0.375
mt-Nd6	1.65×10^{-7}	0.416	4.39×10^{-6}	0.524

Figure 6−figure supplement 1. RBC Progenitor Module Scoring (A) and volcano plot for differentially expressed genes contrasting reclustered RBC progenitor cell clusters erythropoietic and T cell cluster (B).

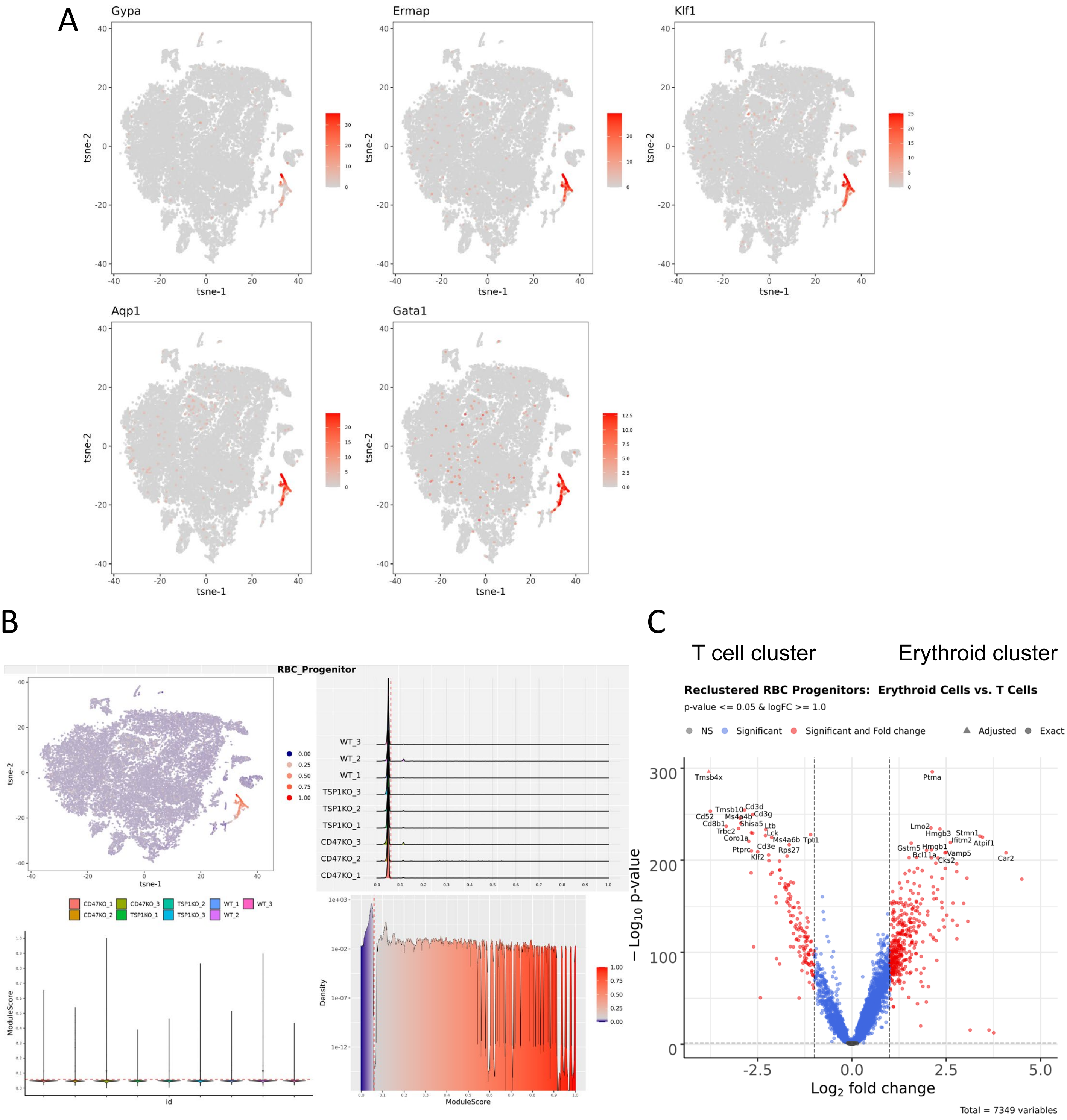

Figure 7−figure supplement 1. Differential expression of erythropoietic, stem cell, and proliferation associated markers in reclustered erythroid and T cell clusters.

Cluster	Gene	% positive cd47$^{-/-}$ cells	% positive thbs1$^{-/-}$ cells	% positive WT cells
Erythroid	Klf1	91.5	84.5	99.1
T cells	Klf1	25.6	21.4	26.0
Erythroid	Aqp1	78.9	83.0	84.5
T cells	Aqp1	32.2	21.4	28.9
Erythroid	Tfrc	45.8	37.9	36.7
T cells	Tfrc	-	-	-
Erythroid	Epor	60.1	56.9	64.4
Erythroid	Ermap	70.7	65.6	64.4
T cells	Ermap	26.4	23.9	20.2
Erythroid	Gata1	80.8	58.6	75.6
Erythroid	Mki67	77.1	69.0	58.9
T cells	Mki67	-	-	-
Erythroid	Kit	80.1	77.6	68.9
T cells	Kit	-	-	-
Erythroid	Xpo1	69.4	56.9	37.8
T cells	Xpo1	21.7	23.9	16.8
Erythroid	Ranbp1	96.7	98.4	91.1
T cells	Ranbp1	54.3	61.5	60.1
Erythroid	Ranbp2	86.7	67.2	48.9
T cells	Ranbp2	46.1	36.8	27.7
Erythroid	Nr3c1	63.5	67.2	41.1
T cells	Nr3c1	44.2	47.9	27.7
Erythroid	Ddx46	81.5	86.2	50.0
T cells	Ddx46	55.0	59.8	33.5
Erythroid	Hba-a1	23.6	6.9	25.6
T cells	Hba-a1	-	-	-
