# Supplementary material for: Differential regulation by CD47 and thrombospondin-1 of extramedullary erythropoiesis in mouse spleen
Source: bioRxiv. 2023 Sep 28:2023.09.28.559992. Preprint. [Version 1] doi: 10.1101/2023.09.28.559992 (PMC10557659; doi:10.1101/2023.09.28.559992)

Banerjee et al Supplemental Data

Supplemental Figure Legends

Supplemental Figure 1. Enlargement of spleen in the absence of CD47 and bulk RNAseq analysis of lin− *cd47*$^{-/-}$ vs WT spleen cells. A) Representative images of spleens from WT, *cd47*$^{-/-}$ and *thbs1*$^{-/-}$ mice. B) volcano plot for differentially expressed genes between lineage-depleted Naïve *cd47*$^{-/-}$ vs WT CD3^{+} spleen cells. Red dots represent significant genes with >2-fold change and p<0.001, and the top 30 genes were labelled. C). GSEA plot showing RBC gene set enrichment and, D) Heat map visualization of the top differentially expressed genes in the RBC gene set.

Supplemental Figure 2. Flow cytometry analysis strategy. The sequential flow cytometry gating is illustrated.

Supplemental Figure 3. Single cell RNA sequence post-filter QC plots. The scatter plots, histograms and violin plots of three samples lineage-depleted of WT, *cd47*$^{-/-}$ and *thbs1*$^{-/-}$ spleen cells are shown. The number of UMI per cell filter was set to exclude cells with < 2000 UMI in nCount RNA. The Feature RNA plot shows the number of genes with non-zero expression detected per cell. Filtering was set to exclude cells having <15% mitochondrial gene expression as presented in the percent mt plot. The log10Genes per UMI plot represents the scores for complexity of the RNA library found in each cell. No filter was set in this row.

Supplemental Figure 4. Cell Type Annotation using SingleR and mRNA expression of the indicated genes. A) The cell types were analyzed using SingleR annotations present in the database and expecting the closet cell types. The Cell type analysis using SingleR was done with two annotation databases, Immgen main cell type annotations and Mouse RNAseq main cell type annotations. B) The distributions of mRNA expression of the indicated genes related to stem cells and erythropoietic cells are shown throughout the 13 clusters.

Supplemental Figure 5. Violin plots of erythropoietic, stem cell, and proliferation associated markers in 13 cell clusters. The distribution patterns of genes related to stem cells, erythropoietic cells, and proliferating cells are shown in all the 13 clusters.

Supplemental Figure 6. Differential expression of erythropoietic, stem cell, and proliferation associated markers in cell clusters 9 and 11. A) Percentages of cells with detectable mRNA expression of the indicated erythropoietic, stem and proliferative markers in cluster 9 and cluster 11 in WT, cd47$^{-/-}$, and *thbs1*$^{-/-}$ spleens. (B) Differential mRNA expression of the nuclear export protein Xpo1 and nuclear pore protein synthesis instructor Ranbp2, which increase erythropoiesis by stabilizing Gata1 in the nucleus, in cluster11 and CD34+ and CD34− subsets of cluster 9 cells.

Supplemental Figure 7. Strategy for reclustering spleen cells that express a gene signature for committed erythroid precursors. A) The top left panel shows the distribution of cells expressing threshold levels of the 4 gene signature in the original TSNE projection. (A) The RBC progenitor module scoring was performed, as module Scores (a.k.a. Signature Scores) calculated for each cell using expression of four genes (Gypa, Ermap, Klf1, and Aqp1). The threshold set manually (red dashed line on subplots) to separate high-scoring cells from low-scoring cells and also expressed by the color (high score cells are dark red and low scores cells are represented as low red). RBC Progenitor Module Scoring Plots show the distributions of module scores in lineage-depleted spleen cells from each mouse, with the threshold score indicated by the dotted red lines. B) cells with positive module scores were reclustered, and a UMAP projection is presented in the top panel. The volcano plot presents differentially expressed genes contrasting the reclustered erythropoietic progenitor cell clusters and the T cell cluster.

Supplemental Figure 1. Enlargement of spleen in the absence of CD47 (A) and bulk RNAseq analysis of lin^{-} *cd47*$^{-/-}$ vs WT spleen cells (B-D)

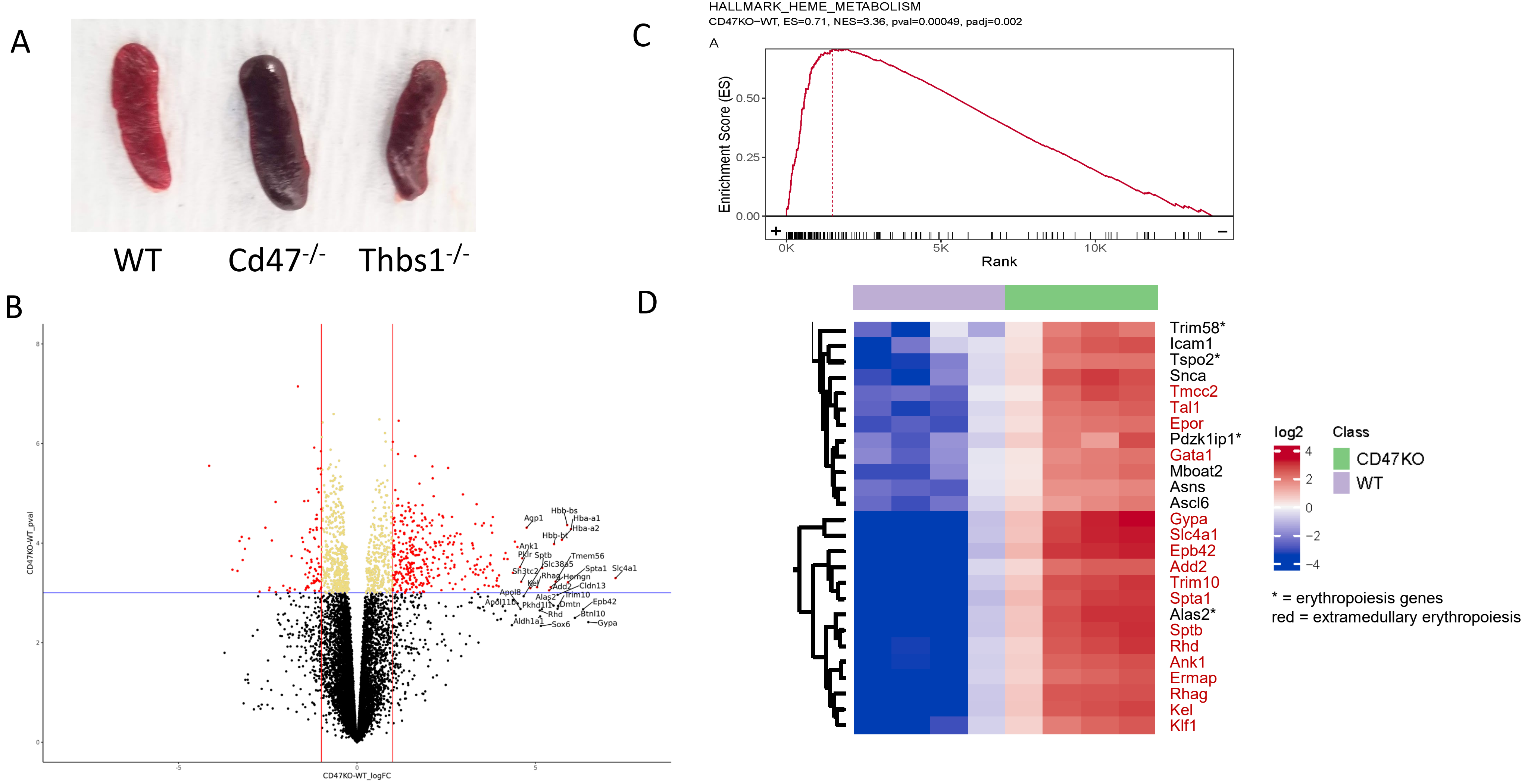

Supplemental Figure 2. Flow cytometry gating

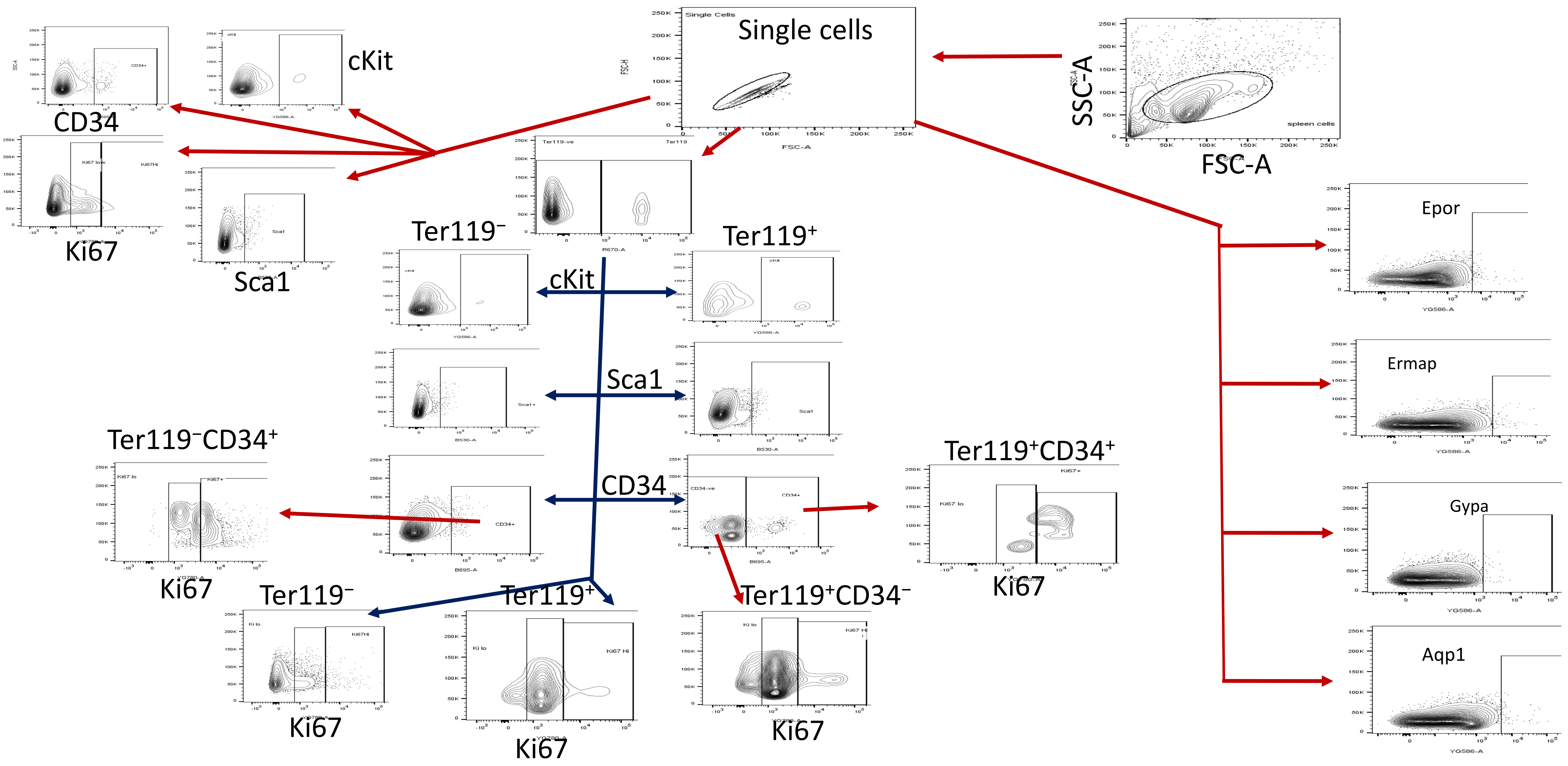

Supplemental Figure 3: scRNAseq Post-Filter QC Plots

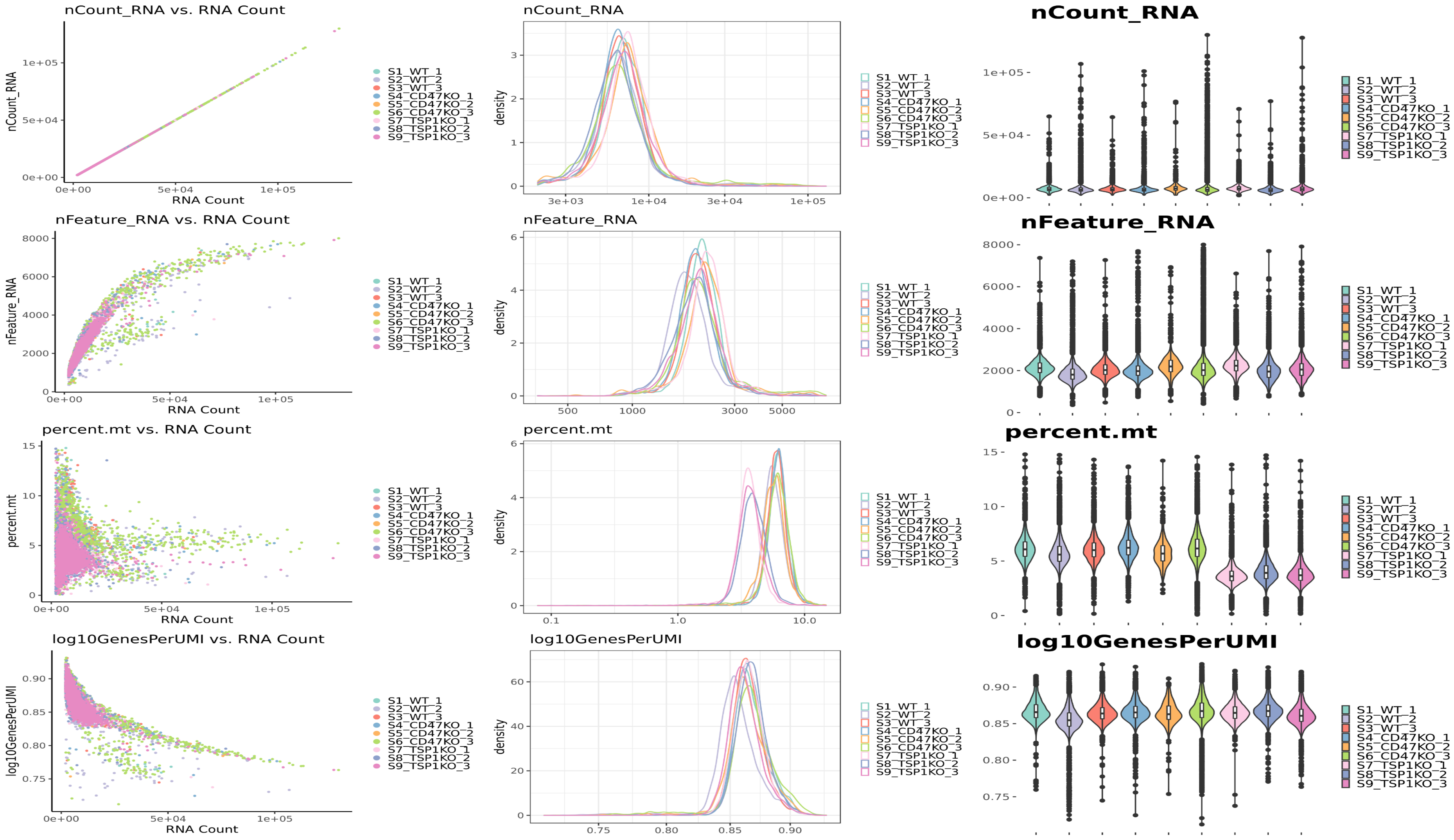

Supplemental Figure 4. Cell Type Annotation using SingleR (A) and mRNA expression of the indicated genes (B).

A

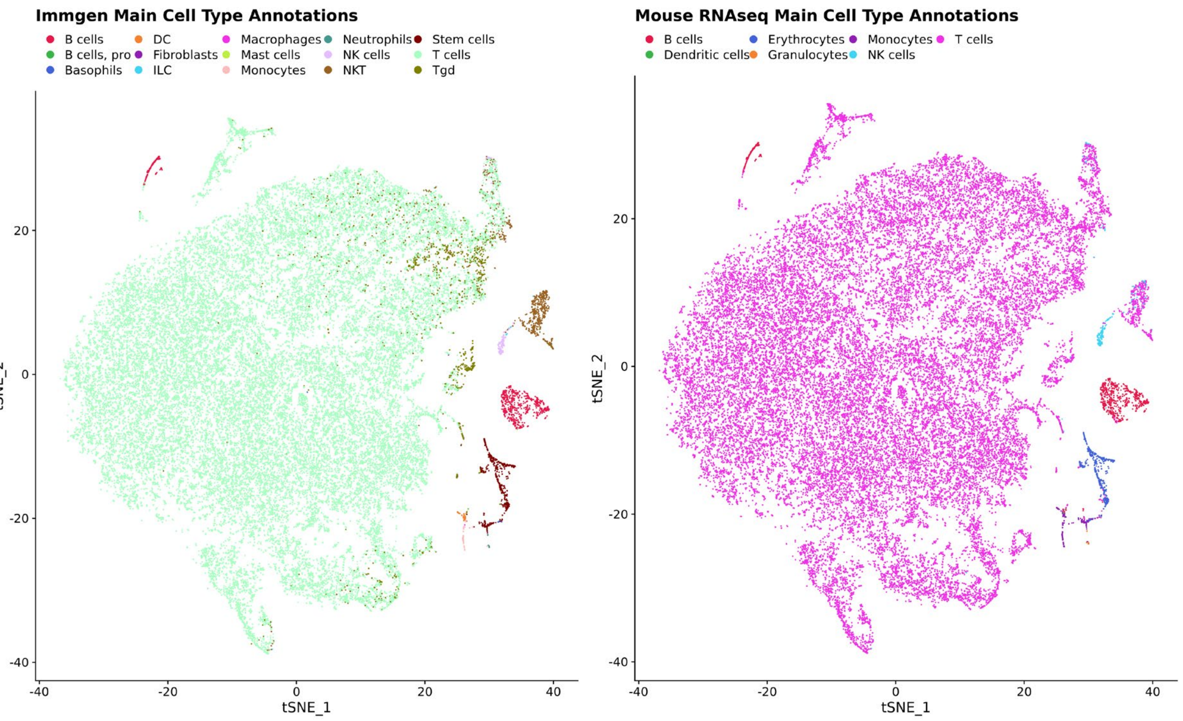

B

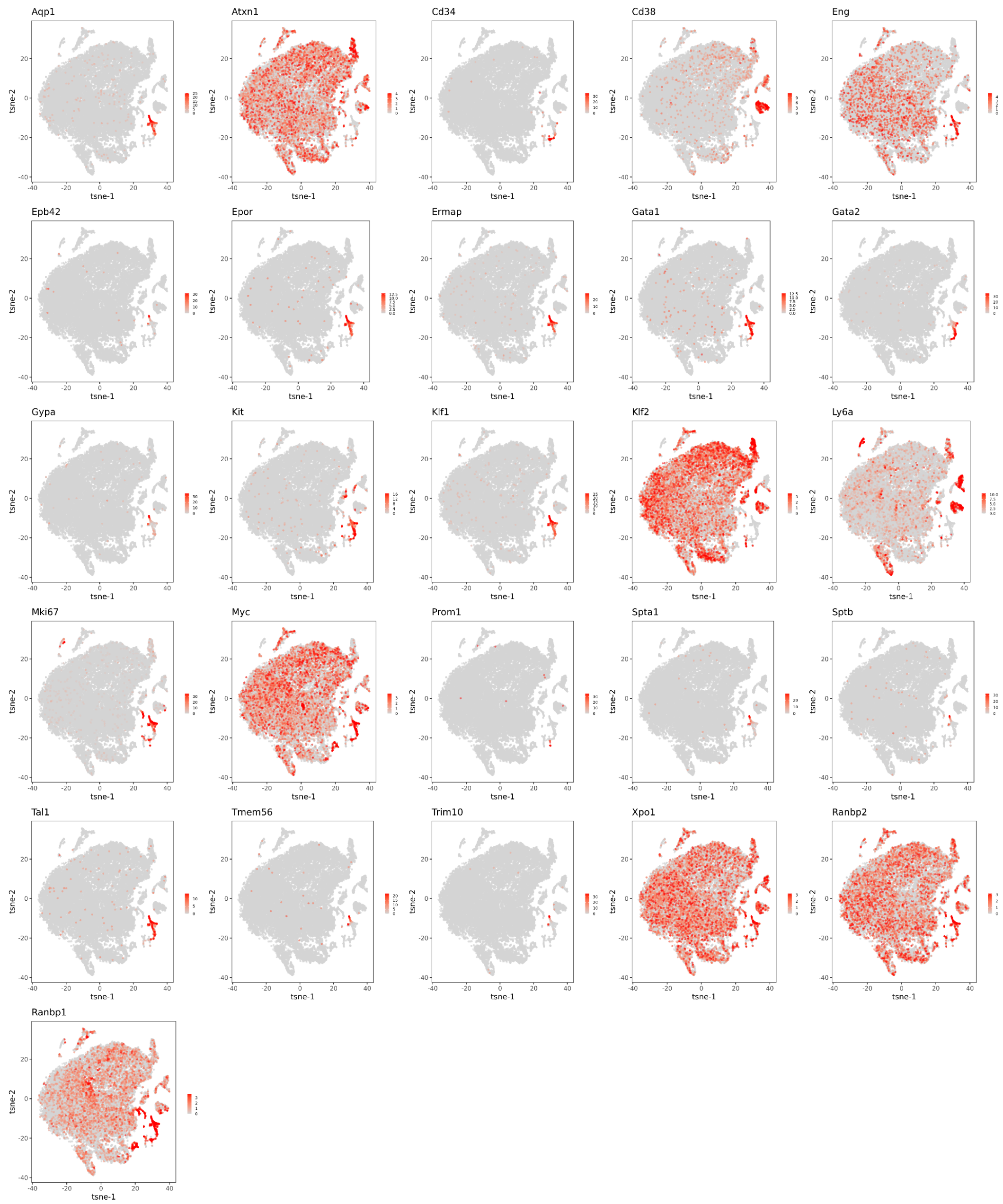

Supplemental Figure 5. Violin plots of erythropoietic, stem cell, and proliferation associated markers in 13 cell clusters

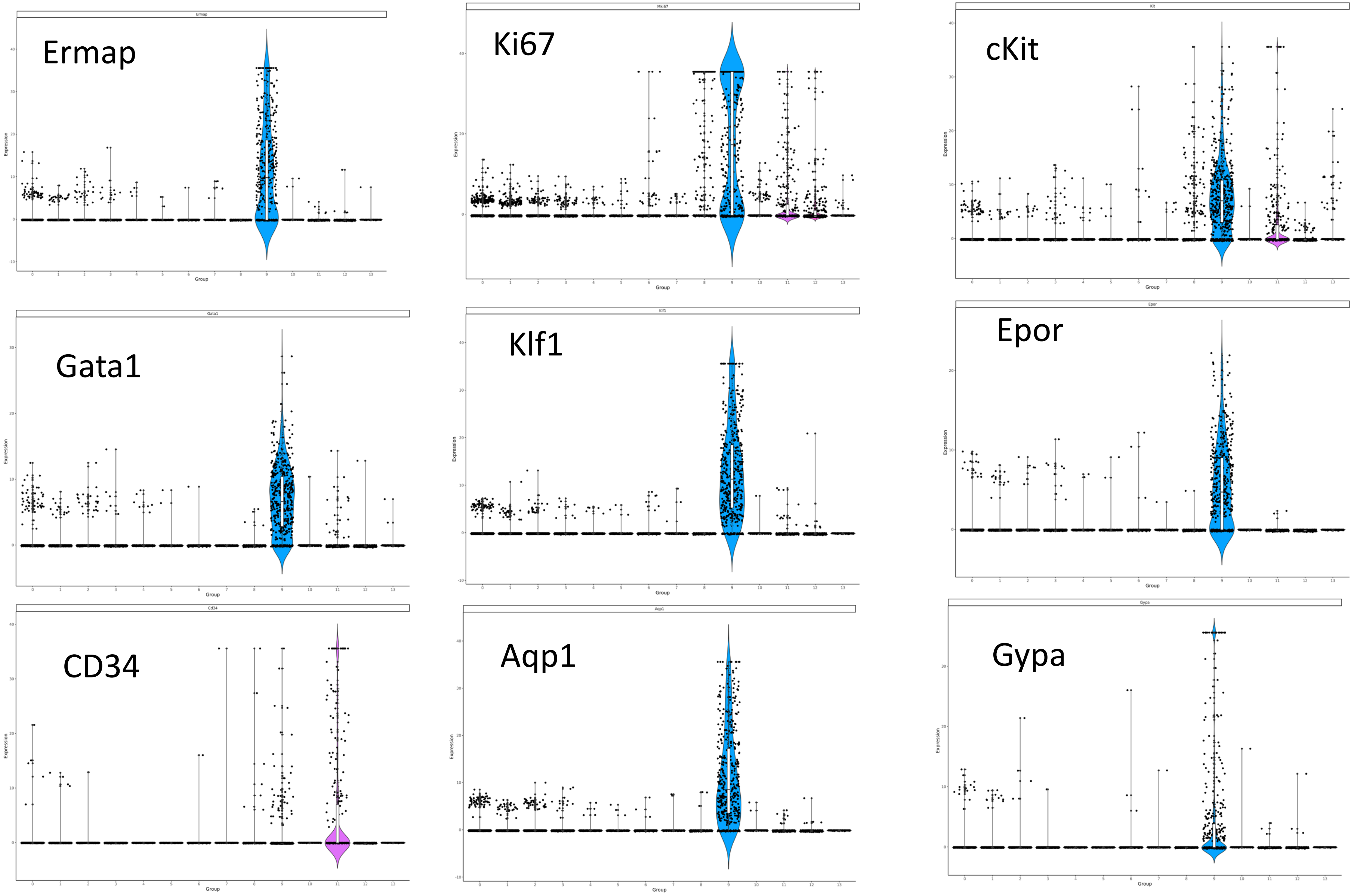

Supplemental Figure 6. Differential expression of erythropoietic, stem cell, and proliferation associated markers in cell clusters 9 and 11.

A

Cluster	Gene	% positive cd47$^{-/-}$ cells	% positive thbs1$^{-/-}$ cells	% positive WT cells
9	Klf1	88	78.5	88.2
9	Aqp1	80.3	76.9	81.7
9	Tfrc	44	35.4	35.5
9	Epor	58.5	47.7	64.5
9	Ermap	66.9	64.6	64.5
9	Gata1	77.8	52.3	74.2
9	Mki67	74.6	63.1	58.1
11	Mki67	41.1	93	23.1
9	Kit	81.7	73.8	66.7
11	Kit	31.3	20	15.4
9	Xpo1	65.8	50.8	36.6
11	Xpo1	38	30.7	24.6
9	Ranbp1	96.1	98.5	91.4
11	Ranbp1	71.8	54.7	69.2
9	Ranbp2	86.3	64.6	49.5
11	Ranbp2	58.9	38.7	35.4
9	Nr3c1	64.1	67.7	43
11	Nr3c1	48.5	64	29.2
9	Ddx46	81	86.2	55.9
11	Ddx46	54.6	50.7	27.7

B

Cell cluster	mRNA	Cd47$^{-/-}$ vs WT		Thbs1$^{-/-}$ vs WT
		p-value	Avg FC	p-value
C9	Xpo1	3.9×10^{-8}	1.25	0.0070
C9 (CD34^{-})	Xpo1	5.9×10^{-8}	1.26	NS
C9 (CD34^{+})	Xpo1	NS		NS
C11	Xpo1	NS		NS
C9	Ranbp2	4.3×10^{-15}	1.45	NS
C9 (CD34^{-})	Ranbp2	7.3×10^{-16}	1.50	NS
C9 (CD34^{+})	Ranbp2	NS		NS
C11	Ranbp2	0.0044	1.20	NS

Supplemental Figure 7. RBC Progenitor Module Scoring (A) and volcano plot for differentially expressed genes contrasting reclustered RBC progenitor cell clusters erythropoietic and T cell cluster (B).

A

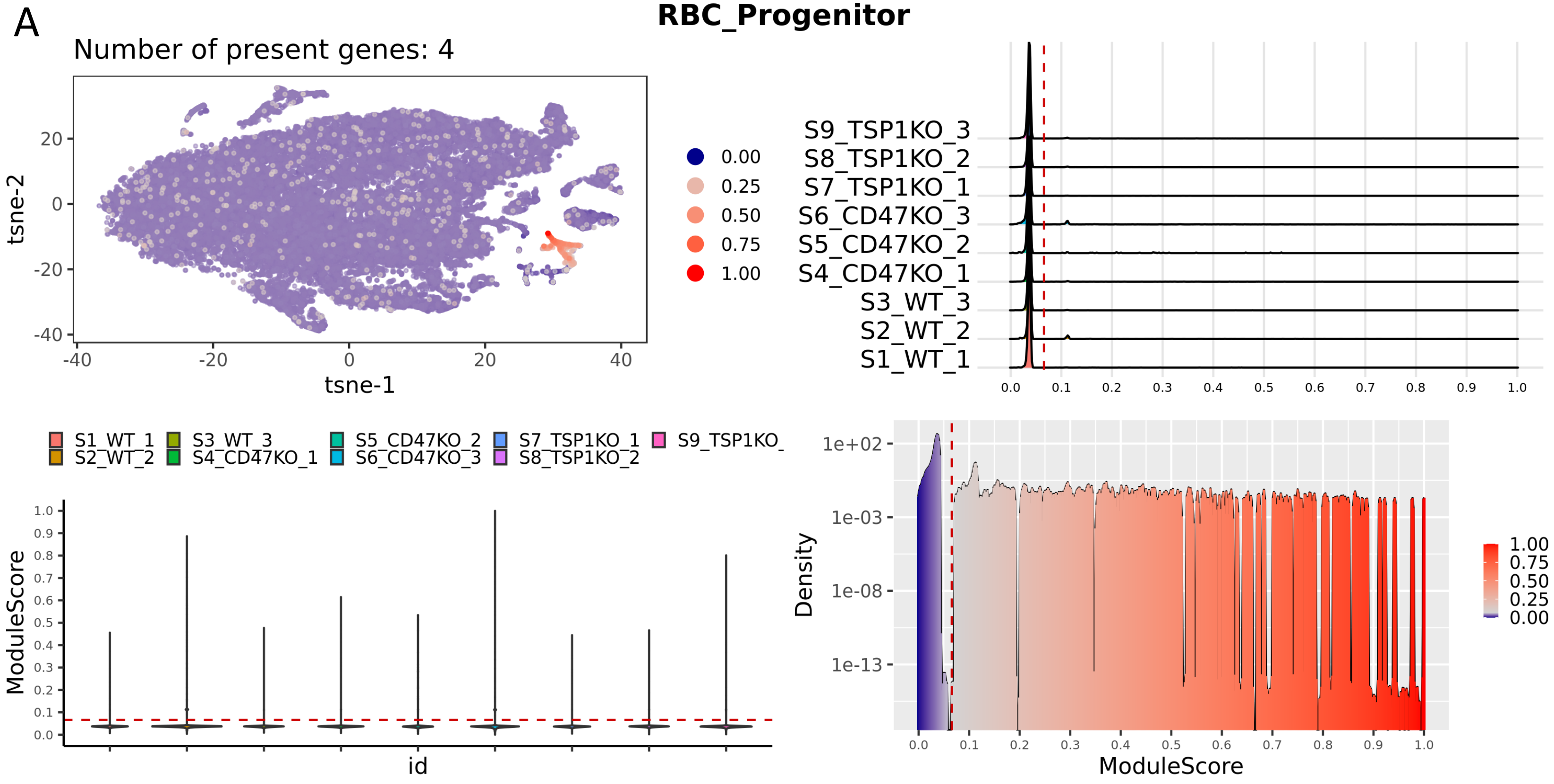

B

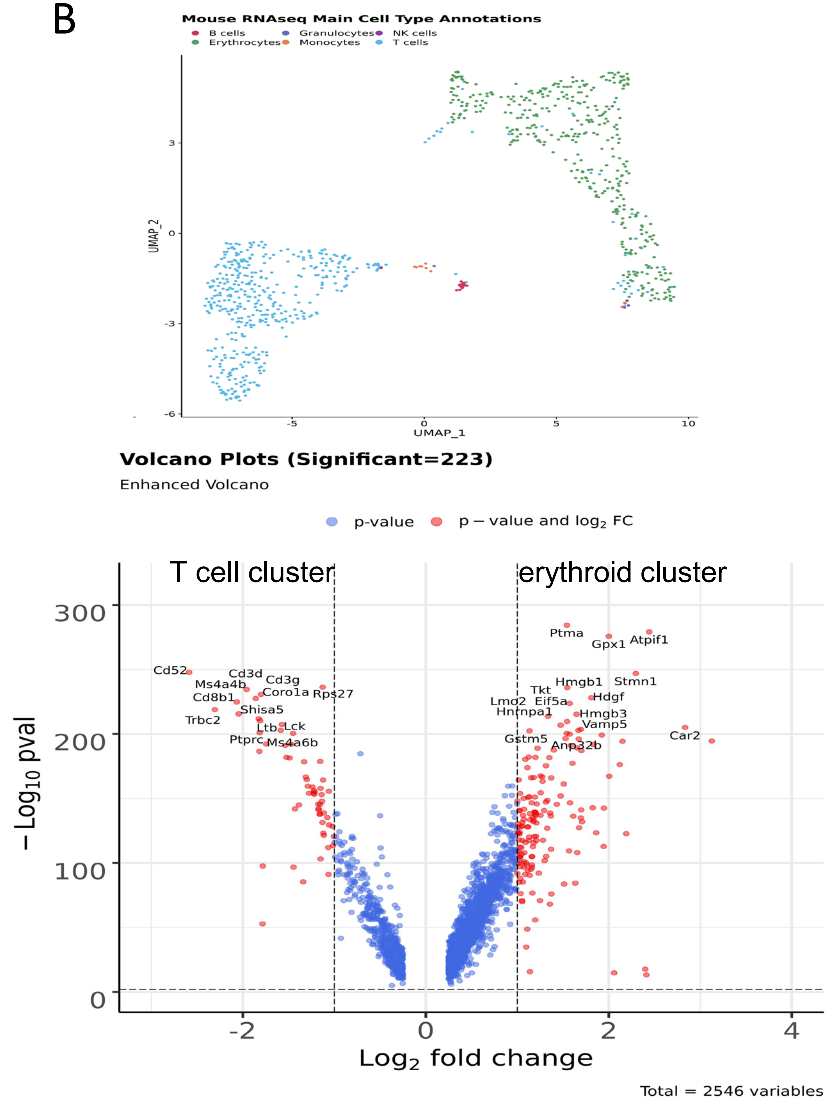

Supplement: Supplementary file 1 [file media-1.pdf]
